# Regulatory insight for a Zn_2_Cys_6_ transcription factor controlling effector-mediated virulence in a fungal pathogen of wheat

**DOI:** 10.1371/journal.ppat.1012536

**Published:** 2024-09-23

**Authors:** Evan John, Callum Verdonk, Karam B. Singh, Richard P. Oliver, Leon Lenzo, Shota Morikawa, Jessica L. Soyer, Jordi Muria-Gonzalez, Daniel Soo, Carl Mousley, Silke Jacques, Kar-Chun Tan

**Affiliations:** 1 Centre for Crop and Disease Management, School of Molecular and Life Sciences, Curtin University, Perth, Australia; 2 Agriculture and Food, Commonwealth Scientific and Industrial Research Organisation, Perth, Australia; 3 School of Biosciences, University of Nottingham, Nottingham, United Kingdom; 4 Université Paris-Saclay, INRAE, UR BIOGER, Thiverval-Grignon, France; 5 Curtin Health Innovation Research Institute, Curtin University, Perth, Australia; Purdue University, UNITED STATES OF AMERICA

## Abstract

The regulation of virulence in plant-pathogenic fungi has emerged as a key area of importance underlying host infections. Recent work has highlighted individual transcription factors (TFs) that serve important roles. A prominent example is PnPf2, a member of the Zn_2_Cys_6_ family of fungal TFs, which controls the expression of effectors and other virulence-associated genes in *Parastagonospora nodorum* during infection of wheat. PnPf2 orthologues are similarly important for other major fungal pathogens during infection of their respective host plants, and have also been shown to control polysaccharide metabolism in model saprophytes. In each case, the direct genomic targets and associated regulatory mechanisms were unknown. Significant insight was made here by investigating PnPf2 through chromatin-immunoprecipitation (ChIP) and mutagenesis approaches in *P*. *nodorum*. Two distinct binding motifs were characterised as positive regulatory elements and direct PnPf2 targets identified. These encompass known effectors and other components associated with the *P*. *nodorum* pathogenic lifestyle, such as carbohydrate-active enzymes and nutrient assimilators. The results support a direct involvement of PnPf2 in coordinating virulence on wheat. Other prominent PnPf2 targets included TF-encoding genes. While novel functions were observed for the TFs PnPro1, PnAda1, PnEbr1 and the carbon-catabolite repressor PnCreA, our investigation upheld PnPf2 as the predominant transcriptional regulator characterised in terms of direct and specific coordination of virulence on wheat, and provides important mechanistic insights that may be conserved for homologous TFs in other fungi.

## Author summary

Fungal pathogens cause large crop losses worldwide and consequently much attention has focused on improving host genetic resistance to diseases. These pathogens use effectors, which require coordinated expression at specific stages of the pathogenic lifecycle, to manipulate the host plant metabolism in favour of infection. However, our understanding of the underlying regulatory components, their direct interactions and their evolutionary origins is lacking. The Pf2 TF-orthologue family underpins virulence and effector gene expression in several fungal phytopathogens, including *Parastagonospora nodorum*. This study provided significant insight into the DNA-binding regulatory mechanisms of *P*. *nodorum* PnPf2, as well as further evidence for its important role in regulating virulence. In the context of crop protection, the Pf2 TFs present opportune targets in major fungal pathogens to screen for direct or indirect inhibitor compounds to suppress virulence and improve disease resistance.

## 1. Introduction

Significant advances have been made in research on the molecular virulence factors underpinning infection by the wheat fungal pathogen *Parastagonospora nodorum*. This fungus produces small secreted effector proteins that interact with host-receptors encoded by dominant susceptibility genes [1,2]. These interactions occur in a gene-for-gene manner that causes ‘effector-triggered susceptibility’ in the host plant, quantitatively affecting the disease which manifests as septoria nodorum blotch. Several effectors acting in this manner have now been identified and characterised for their role in virulence [3–7]. These studies have also described a consistent pattern: the expression of these genes is maximal two to four days after infection and then declines. Furthermore, expression levels can vary depending on the presence or absence of their matching wheat receptors, as well as by epistasis, whereby one effector gene causes suppression of another [8–10]. Relatively little is known concerning the mechanisms governing the effector gene regulation. In particular, are there common or distinct regulatory pathways involved? Do these components specifically control effector gene expression, or co-regulate other metabolic and developmental pathways? New knowledge in this area could present suitable targets to suppress for disease control by screening for inhibitor compounds that target such signalling/regulatory components.

Many fungi possess the Zn_2_Cys_6_ transcription factor (TF) Pf2 which has been associated with the regulation of effector gene expression. One example is the AbPf2 orthologue in *Alternaria brassicicola* that is critical for virulence on *Brassica* spp. [11]. Gene deletion of *AbPf2* resulted in the down-regulation of effector like genes, as well as putative cell-wall degrading enzymes. In *P*. *nodorum*, at least two key effector genes, *ToxA* and *Tox3*, require PnPf2 to be expressed [12]. An RNA-seq analysis also revealed that PnPf2 regulates many more putative effectors, carbohydrate-active enzymes (CAZymes), peptidases, other hydrolases and nutrient transporters [13]. The PtrPf2 orthologue in *Pyrenophora tritici-repentis* controls *PtrToxA* expression and virulence on wheat, much like the homologous *ToxA* gene in *P*. *nodorum* [12]. In *Leptosphaeria maculans*, the causal agent of blackleg disease on *Brassica* spp., the orthologue LmPf2 also regulates several effector genes, including *AvrLm4-7*, *AvrLm6*, *AvrLm10A* and *AvrLm11*, as well as CAZyme expression [14].

Pf2 orthologues can be traced across several Ascomycota fungal lineages including the Dothideomycetes, Leotiomycetes and Sordariomycetes [15]. Deletion of the corresponding genes in the plant pathogens *Botrytis cinerea*, *Fusarium* spp., *Magnaporthe oryzae* and *Zymoseptoria tritici* all suppressed fungal virulence as well as their capacity to utilise alternative carbon sources [16–19]. Analogous regulatory roles pertaining to carbon utilisation have been described in the saprophytic fungi *Neurospora crassa* and *Trichoderma reesei* [20,21]. In *N*. *crassa*, the putative orthologue Col-26 is a critical component within a signalling-network that involves the carbon-catabolite transcriptional repressor Cre-1 and responds to glucose availability to control the expression of CAZymes for plant cell-wall degradation [22–24]. A strong correlation has also been observed between CAZyme gene content and plant-pathogenic lifestyles [25], yet whether conserved or divergent regulatory pathways control their expression is underexplored.

There are some key factors to be established among the Pf2 orthologues. Which DNA-regulatory elements are bound? Are Pf2-regulated genes directly targeted or is their expression modulated indirectly, by other transcriptional regulators? The research presented herein provides critical insight using the *P*. *nodorum*-wheat pathosystem as a model. A direct regulatory role in effector/CAZyme expression is identified, and several other TFs are independently characterised in connection with PnPf2 regulation.

## 2. Results

### 2.1. PnPf2 harbours Zn_2_Cys_6_ domains and localises to the nucleus

**Fig 1:**
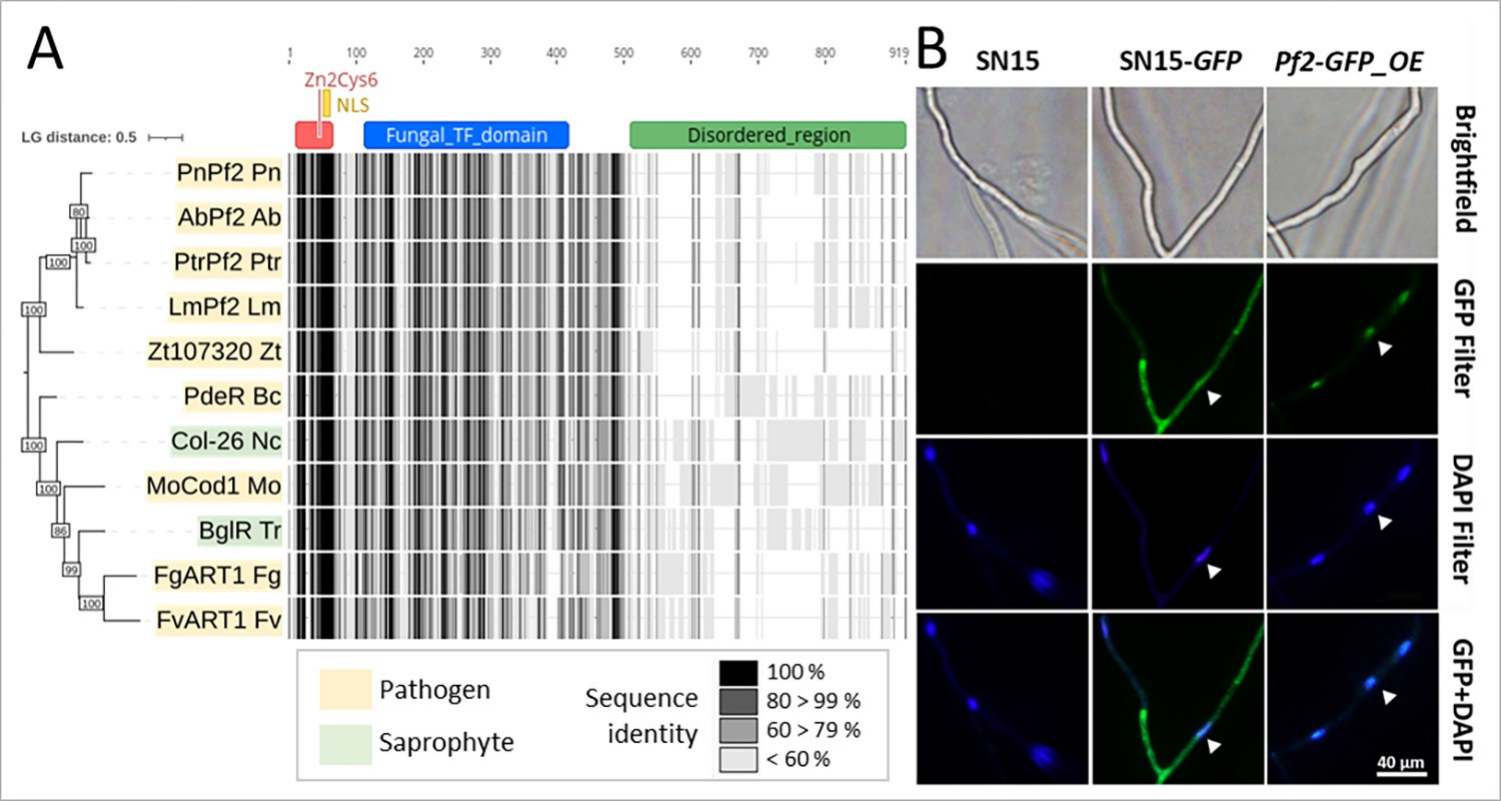
PnPf2 domain overview, phylogeny and cellular localisation. **A)** A maximum likelihood tree and alignment for functionally investigated PnPf2 orthologues in the literature. Adapted from John et al. [15] (https://creativecommons.org/licenses/by/4.0/ - non PnPf2 orthologues excluded from current version). Bootstrap values for 100 replicates are indicated. Domains identified in the 652 amino acid PnPf2 protein are indicated above, including the N-terminal Zn_2_Cys_6_ DNA binding domain in red (Interpro IPR001138), the nuclear localisation signal (NLS) in yellow and the fungal transcription factor domain in blue (Interpro IPR007219). The C-terminal is poorly conserved corresponding to a disordered region (green). Species abbreviations, Pn; *Parastagonospora nodorum*, Ab; *Alternaria brassicicola*, Ptr; *Pyrenophora tritici-reprentis*, Lm; *Leptosphaeria maculans*, Zt; *Zymoseptoria trititci*, Bc; *Botrytis cinerea*, Nc; *Neurospora crassa*, Mo; *Magnaporthe oryzae*, Tr; *Trichoderma reesei*, Fg; *Fusarium graminearm*, Fv; *Fusarium verticillioides*. **B)** Epifluorescence microscopy depicting nuclear localisation of a GFP-tagged PnPf2 translational fusion specific to the *Pf2-GFP_OE* overexpression strain, in contrast to the wildtype (SN15) and the positive control strain expressing cytoplasmic GFP (SN15-*GFP*). Arrows indicate the corresponding locations of fungal nuclei under the respective filters determined by DAPI staining of a germinated pycnidiospore.

Prior to functional investigation we sought to identify individual conserved features related to TF activity across the 652 amino acid (a.a) PnPf2 protein. A conserved Zn_2_Cys_6_ DNA binding domain was located N-terminally at a.a 9 to 54 with an overlapping nuclear localisation signal (NLS) (KKGPKGSR; a.a 51 to 58) (**Fig 1A**). A conserved ‘fungal TF domain’ was identified from a.a 223 to 294 within a conserved ‘middle homology region’ (a.a 104 to 320). These features are frequently observed in Zn_2_Cys_6_ TFs and have been linked to the modulation of TF activity [26,27,15]. A structurally disordered domain, typically associated with post-translational modifications and intermolecular interactions [28], was also identified at the C-terminus of PnPf2 and is poorly conserved in characterised orthologues. Nuclear localisation of the C-terminally tagged PnPf2-GFP fusion protein was also observed (**Fig 1B**). Together, these observations suggest PnPf2 possesses typical features of DNA-binding Zn_2_Cys_6_ TF activity [26].

### 2.2. Two direct PnPf2 target motifs are associated with gene-regulation

A chromatin immunoprecipitation (ChIP) analysis was used to define PnPf2-DNA binding sequences. Despite efforts, sufficient fungal material could not be obtained under early infection conditions *in planta* where PnPf2 is maximally expressed. Instead, the *in vitro* culture conditions used previously for RNA-seq [13] were replicated. Strains expressing a 3x haemagglutinin (HA) tagged PnPf2-HA fusion protein under both the native promoter (*Pf2-HA*) and through overexpression (*Pf2-HA_OE*) were generated. The latter was included as *PnPf2* expression is comparably lower *in vitro* [13] and would allow more potential binding sites to be captured through ChIP. Both *Pf2-HA* and *Pf2-HA_OE* were phenotypically comparable despite the difference in *PnPf2* expression, and retained PnPf2 virulence-regulatory function in contrast to a *pf2-HA_KO* deletion control (**S1 Text**). Both stains were subject to a ChIP-seq analysis, which identified a number of ‘summits’ within enriched ‘peak’ regions. These summits correspond to the best estimate of DNA binding loci within peaks [29] and were only retained for *Pf2-HA* and *Pf2-HA_OE* if (i) they were detected in two biological replicates for each strain and (ii) no overlapping peaks were detected from the *pf2-HA_KO* control dataset (**S1 Text**). A total of 760 summits across 586 peaks were obtained from the *Pf2-HA* dataset. Of these, 538 were reproducible in the *Pf2-HA_OE* dataset which comprised 2081 summits across 1536 peaks (**S1 Text; S1 File**). A quantitative PCR (qPCR) analysis was then undertaken to independently assess summit enrichment, comparing the *Pf2-HA* and *Pf2-HA_OE* samples to the *pf2-HA_KO* control. Fold-enrichment values across a number of loci strongly correlated with ChIP-seq summit -Log_10_(Q-values), a proxy measure for PnPf2-DNA binding affinity, in both the *Pf2-HA* (P < 0.01 with Pearson’s r = 0.77) and *Pf2-HA_OE* (P < 0.01 with Pearson’s r = 0.74) datasets (**Table 1**). The high reproducibility across separate methodologies provided confidence in the robustness of ChIP-seq summit calls.

**Table 1:** Verification of enriched ChIP-seq summits by quantitative PCR ^A^.

Target locus	*Pf2-HA* ^B^		*Pf2-HA_OE* ^C^	
	qPCR Enrichment	Summit Q-values	qPCR Enrichment	Summit Q-values
*Actin* exon (-)	1.0	-	1.0	-
*TrpC* terminator (-)	0.9	-	1.2	-
*ToxA* promoter	0.9	-	1.4	6.5
*Tox3* promoter	2.2	325.9	3.7	550.7
*Tox1* promoter	0.8	-	3.6	121.6
SNOG_03901 promoter	1.2	5.4	1.2	37.7
SNOG_04486 promoter	0.9	9.5	4.4	191.2
SNOG_12958 promoter	2.2	236.3	5.7	252.3
SNOG_15417 promoter	2.1	78.0	2.7	139.3
SNOG_15429 promoter	3.5	180.4	5.9	492.9
SNOG_15429 exon (-)	0.9	-	1.7	-
SNOG_16438 promoter	0.7	-	3.4	125.4
SNOG_20100 promoter	1.5	21.5	3.9	160.9
SNOG_30077 promoter	1.1	-	5.4	231.0

^A^ The qPCR values represent fold-enrichment vs the *pf2-HA_KO* control strain and the summit values represent ChIP-seq -Log_10_(Q-values). Target loci listed with (-) were included as qPCR negative controls where no ChIP-seq summit was predicted. Linear regression was used to assess correlation between respective fold-enrichment and Q-values. ^B^ Significantly correlated values (P < 0.01) based on Pearson’s correlation (*r* = 0.77). ^C^ Significantly correlated values (P < 0.01) based on Pearson’s correlation (*r* = 0.74).

**Fig 2:**
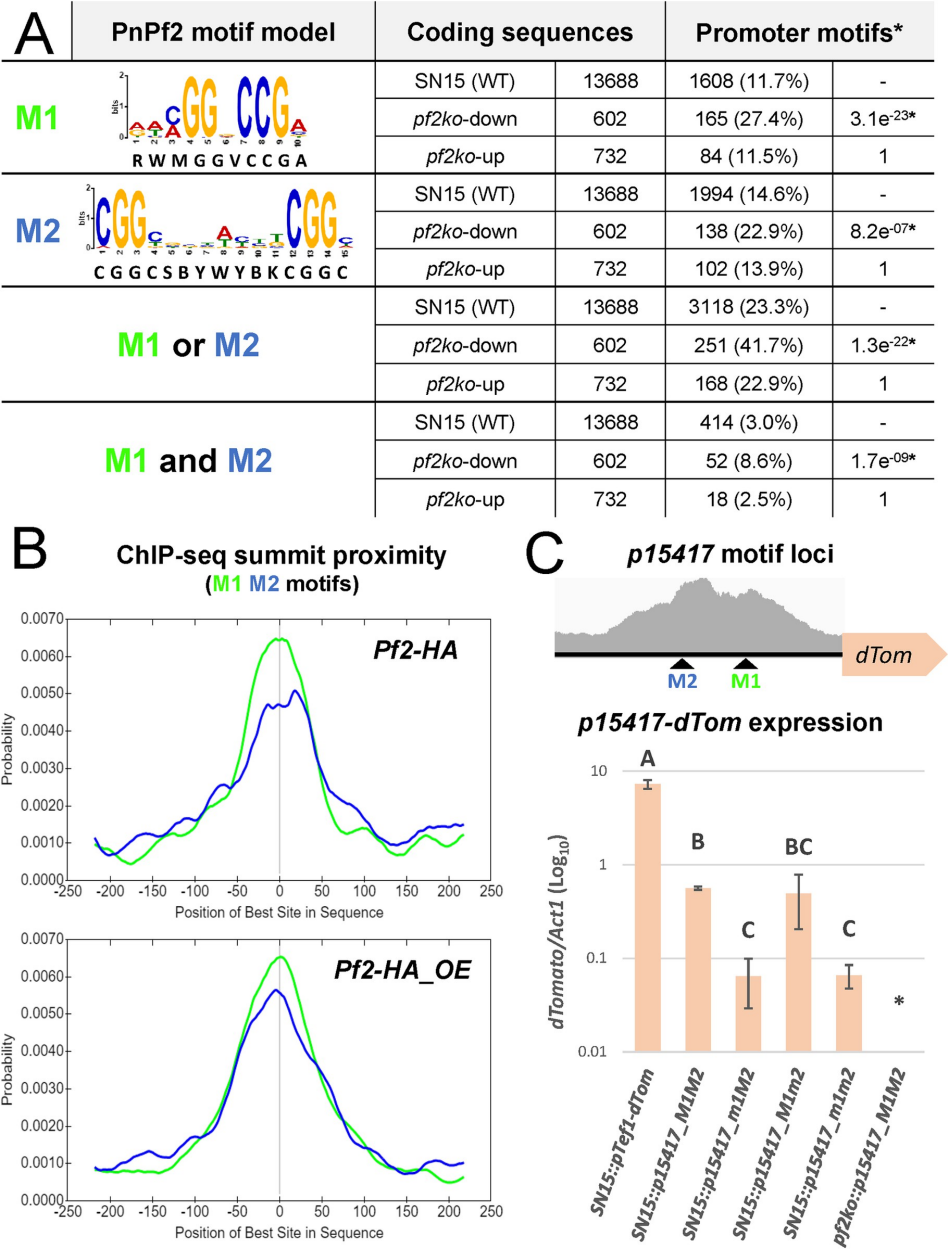
Identification of PnPf2 regulatory element motifs. **A)** The M1 motif (5’-RWMGGVCCGA-3’) and M2 motif (5’-CGGCSBYWYBKCGGC-3’) were modelled from the merged set of *Pf2-HA* and *Pf2-HA_OE* sample ChIP-seq peak regions. Their detection (≥ 1 occurrence, alone or in combination) in coding-sequence promoters of PnPf2 positively (*pf2ko*-down) or negatively (*pf2ko*-up) regulated gene promoters [13] are indicated relative to all SN15 (wildtype) promoters. *The P_adj_ values are indicated in the final column and reflect the test for significant enrichment (Fisher’s test with Bonferroni P_adj_ < 0.01), where both motifs were enriched in the *pf2ko*-down promoters relative to SN15. **B)** The position of motif occurrences relative to ChIP-seq summits, demonstrating their higher likelihood at close proximity to the best estimate of PnPf2-DNA binding loci. **C)** Gene expression analysis assessing the effect of M1 and M2 motif mutation in *P*. *nodorum in situ*. The motif loci within a ChIP-seq peak in the *SNOG_15417* gene promoter region (*p15417*) are depicted. The *dTomato* reporter gene was fused to a constitutive promoter control (*pTef1-dTom*) or the *SNOG_15417* gene promoter (*p15417_M1M2*) in the wildtype (WT) SN15 background. Motifs were also mutated at respective ‘CGG’ triplets, alone or in combination (*p15417_m1M2*, *p15417_M1m2* and *p15417_m1m2*). Gene expression was measured by qRT-PCR using cDNA extracted under ChIP culture conditions. Letters indicate statistically distinct groupings by ANOVA with Tukey’s-HSD (P<0.05). Error bars indicate standard deviations of three biological replicates. *dTomato expression was not detectable under the *p15417_M1M2* promoter in the *pf2ko* mutant background.

Previous RNA-seq differential-expression analyses had identified an enriched consensus motif (5’-WMGGVCCGAA-3’) in the promoter regions of both AbPf2 and PnPf2-regulated genes [11,13]. Despite harbouring the typical ‘CGG’ Zn_2_Cys_6_ binding triplet [26], an interaction with PnPf2 was not observed in a heterologous system, indicating regulatory cofactors may be required [30,13]. Here, two enriched motifs were identified from the merged *Pf2-HA* and *Pf2-HA_OE* peaks (**Fig 2A**). The first motif designated as M1 (5’-RWMGGVCCGA-3’) closely matches the consensus motif from AbPf2 and PnPf2-regulated gene promoters [11,13]. The second motif designated as M2 (5’-CGGCSBYWYBKCGGC-3’) is novel for PnPf2, encompassing two copies of the canonical ‘CGG’ Zn_2_Cys_6_ binding triplets [26], separated by eight nucleotides. Interestingly, M2 matches the AmyR regulatory response element that was modelled in *A*. *nidulans* [31]. Both M1 and M2 are distributed in close proximity to ChIP-seq summits suggesting they accurately reflected DNA-binding loci (**Fig 2B**).

The previous RNA-seq analysis had defined genes positively or negatively-regulated by PnPf2 from their expression changes in the *PnPf2*-deletion mutant *pf2ko* relative to wildtype SN15 [13] (**S1 File**). M1 and M2 motif frequencies were then assessed across the promoters (defined in materials and methods) of PnPf2 positively regulated (i.e. *pf2ko*-down) or negatively regulated (i.e. *pf2ko*-up) genes in comparison to all SN15 coding sequences. Both M1 and M2 were significantly enriched for *pf2ko*-down genes alone and in combination (M1 and M2; M1 or M2) but not *pf2ko*-up genes (**Fig 2A**). This indicates both motifs correspond to cis-regulatory elements that induce, rather than repress, gene expression. Motif orientation did not appear to be a major factor. Across the 602 *pf2ko-*down gene promoters, M1 was detected 95 times forward vs 105 times in reverse orientation; M2 occurred 69 times in forward and 79 times in reverse orientation.

We were unable to confirm a direct interaction with M1 and M2 motifs via electrophoretic mobility shift assay using heterologously expressed PnPf2 due to solubility issues arising from intrinsic protein properties. Previously, we had investigated PnPf2 binding with a motif similar to M1 using a yeast-one-hybrid approach [13]. Since ChIP-seq enabled us to refine this motif, we sought to re-assess this interaction, as well as the newly characterised M2 motif, with PnPf2 through yeast-one-hybrid. Single copies (M1^x1^/M2^x1^) and tandem multi-copies (M1^x2^/M2^x2^ & M1^x3^/M2^x3^) of the respective motifs were used as bait sequences. We found that both M1 and M2 motifs presented as either a single or as three tandem copies were recognised by an unknown endogenous yeast TF(s) leading to auto-transactivation (**S1 Fig**). However, two tandem copies did not lead to auto-activation but interactions with PnPf2 was not observed (**S1 Fig**). Therefore, we were unable to confirm a direct interaction with PnPf2 *in vivo* through yeast-one-hybrid. Instead, we sought a novel approach to detect the specific PnPf2-motif interactions *in situ*, where coregulatory factors exist that are potentially absent in the yeast heterologous system. A positively regulated gene promoter (*SNOG_15417*) harbouring M1 and M2 was fused to the *dTomato* reporter gene. Integration of the construct at a predefined genomic locus in the SN15 background permitted evaluation of the reporter-gene expression in the resultant strain (*p15417_M1M2*) in comparison with strains where the CGG triplets in M1 and/or M2 had been substituted (*p15417_m1M2*, *p15417_M1m2* and *p15417_m1m2*). Significantly reduced *dTomato* expression was observed in the strains where M1 had been mutated. Furthermore, *dTomato* expression was not detected if *p15417_M1M2* was used in the *pf2ko* background, providing further evidence it is a direct PnPf2 target (**Fig 2C**). However, no significant expression change was detected where only the M2 motif was mutated indicating it was not an important regulatory determinant for *SNOG_15417*.

### 2.3. PnPf2 directly targets genes associated with the pathogenic lifestyle of *P*. *nodorum*

**Fig 3:**
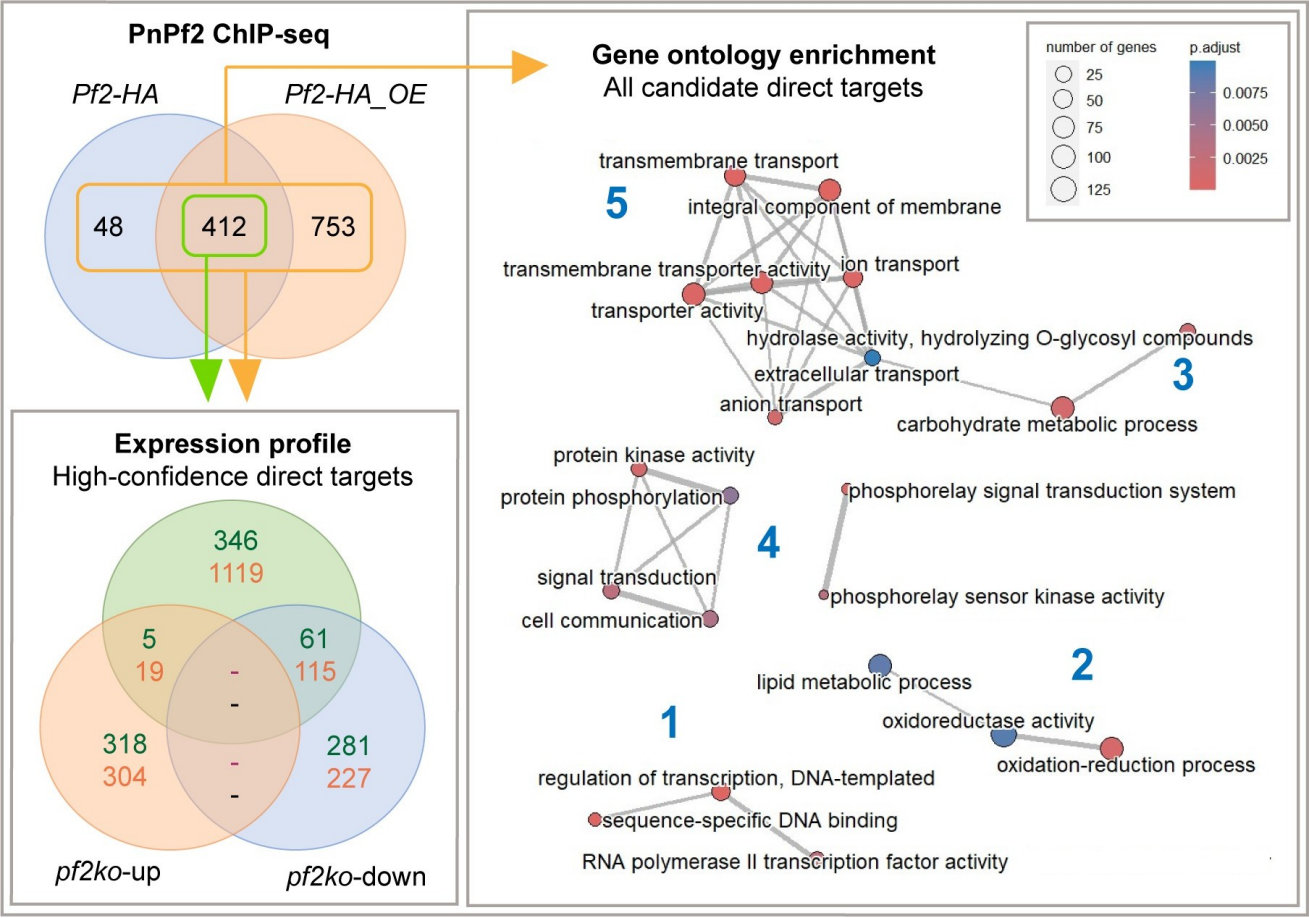
Gene expression and gene-ontology (GO) association of PnPf2 direct targets identified by ChIP-seq from the *Pf2-HA* and *Pf2-HA_OE* ChIP-seq datasets. The 412 high-confidence PnPf2 targets (both datasets—green) and the 1253 total targets (either dataset–orange) were compared to their expression profile (bottom-left panel) in *pf2ko* relative to SN15 [13]. Numbers correspond to significantly up (*pf2ko-up*) or down-regulated genes (*pf2ko-down*) under *in vitro* growth conditions used for ChIP-seq. In both cases far greater binding overlap was observed for *pf2ko-down* than *pf2ko-up*, indicating positive regulation is likely to occur if functional binding takes place. Significantly-enriched GO terms among all PnPf2-targeted genes are also presented (right panel). Bubble sizes are proportionate to gene counts, colours to the enrichment test *P* values and the line width between bubbles to the total shared terms. Numbers in blue indicate connected gene networks representing transcription factors (1), redox molecules (2), carbohydrate-active enzymes (3), cell-signalling molecules (4), and trans-membrane transporters (5). The GO-enrichment plot is also provided for the high-confidence dataset (**S3 Fig**).

Genes with a ChIP-seq promoter summit, considered putative PnPf2 targets, were cross-referenced with the *pf2ko* RNA-seq data analysis (**S1 File**). There were 412 high-confidence targets identified, defined as those with a promoter summit in both the *Pf2-HA* and *Pf2-HA_OE* datasets(**Fig 3**). Of these, 61 genes are PnPf2 positively-regulated in contrast to 5 negatively-regulated genes under the same *in vitro* conditions used for ChIP-seq. This indicates PnPf2 functions mainly as a positive regulator of gene expression, rather than a repressor. A similar pattern was still observed for the 1213 genes with a promoter summit in either ChIP-seq dataset, with 115 positively-regulated by PnPf2 and 19 negatively-regulated (**Fig 3**). For the remaining direct PnPf2 targets with no significant expression change in *pf2ko*, it is possible additional coregulatory factors are absent under the conditions tested.

The characterised effector genes in *P*. *nodorum* SN15, *ToxA*, *Tox1*, *Tox3* and *Tox267* [7], as well as 15 candidate effectors [32] with reduced expression in *pf2ko* were then examined for direct regulation by PnPf2. We also considered 12 additional candidates which, like *ToxA*, are poorly expressed *in vitro* but significantly reduced in *pf2ko* under *in planta* conditions (detailed in **S1 File**), representing potential PnPf2 targets that require extra co-regulatory factors during infection. Of the 31 gene promoters examined, evidence for direct regulation was identified for 11 (**Table 2**). In the bidirectional *Tox3* promoter, two distinct ChIP-seq summits were identified (**S2 Fig**). Both the upstream gene (i.e. *SNOG_08982*, encoding a protein disulphide-isomerase) and *Tox3* are positively regulated by PnPf2 (**S1 File**). A ChIP-seq summit was also identified in the *Tox1* promoter, but only from the *Pf2-HA_OE* dataset (**S2 Fig**). Unlike Tox3, Tox1 necrosis-inducing activity is still detected in the *pf2ko* background [12], indicating the summit may represent a regulatory element quantitatively, but not completely, affecting *Tox1* expression. The *ToxA* gene is only expressed during infection but in a PnPf2-dependent manner. A weak summit was observed in the *ToxA* promoter despite multiple instances of the M1 motif, suggesting another coregulatory factor(s) is required to facilitate PnPf2-DNA binding that was absent under the ChIP-seq experimental conditions. No distinct PnPf2 summit was observed in the promoter of *Tox267*, whose expression is not significantly altered in the *pf2ko* mutant, although two instances of M1 were identified >1000 bp upstream (**S2 Fig**).

**Table 2:** ChIP-seq summit and motif distribution of effector-like genes directly targeted by PnPf2 with *pf2ko* reduced expression^A^.

Gene ID	Summit loci		Motif loci		*pf2ko* -RNA-seq	Protein length	Protein annotation	Annotated homologues^B^
	*Pf2-HA*	*Pf2-HA_OE*	M1	M2				
*Tox3*	-185;-753	-189; -760	-680	-724; -713; -981	*iv–*down *ip–*down			
SNOG_13722	-663	-668	-	-	*iv–*down *ip–*same	136	IPR010829 (Cerato-platanin); IPR009009 (RlpA-like protein, double-psi beta-barrel domain)	*Ds*, *Cb*, *Pf*, *Psf*, *Pt*, *Rc*, *Zb*, *Zt*
SNOG_20100	-708; -1294	-697; -1287	-70; -1303	-1304; -695	*iv–*down *ip–*down	71	-	-
SNOG_08150	-	-204	-206	-196	*iv–*same *ip–*down	124	-	-
SNOG_12218	-	-406	396	-543	*iv–*down *ip–*same	209	-	*Aa*, *Bo*, *Bs*, *Bv*, *Bz*, *Pt*, *Ptr*
SNOG_12449	-	-179	-	-984	*iv–*down *ip–*same	113	-	*Bm*, *Bo*, *Bs*, *Bv*, *Bz*
SNOG_16438	-	-413	-507; -1241	-674	*iv–*same *ip–*down	138	-	*Bm*, *Pt*
*ToxA*	-	-394	-216; -366; -409	-1330	*iv–*same *ip–*down	178	IPR021635 (Proteinaceous host-selective toxin ToxA)	*Bs*, *Ptr*
*Tox1*	-	-197; -578	-598	-	*iv–*down *ip–*down	117	IPR044057 (Tox1, chitin binding-like domain)	-
SNOG_30077	-	-610	-609	-	*iv–*same *ip–*down	67	-	-
SNOG_30352	-	-189	-	-	*iv–*down *ip–*same	80	-	-

^A^ Genes annotated as effectors [32] with significantly reduced expression based in RNA-seq analysis in vitro (*iv*—down) or in planta (*ip*–down) in the *pf2ko* mutant [13]. Classed as PnPf2 direct-targets based on ChIP-seq promoter summit(s). Relative position provided along with putative PnPf2 target-motif loci

^B^ Homologues were identified in the respective Uniprot records for: *Bm; Bipolaris maydis*, *Bo; Bipolaris oryzae*, *Bs; Bipolaris sorokiniana*, *Bv Bipolaris victoriae*, *Bz*, *Bipolaris zeae*, *Cb; Cercospora beticola*, *Pf; Passalora fulva*, *Psf; Pseudocercospora fijiensis*, *Pt; Pyrenophora teres*, *Ptr; Pyrenophora tritici-repentis*, *Rc; Ramularia collo-cygni*, *Zb; Zymoseptoria brevis*, *Zt; Zymoseptoria tritici*

A gene-ontology (GO) enrichment analysis was conducted to identify major functional gene classes that are directly regulated by PnPf2. Five distinct groups representing TFs, redox molecules, CAZymes, cell-signalling molecules and nutrient transporters were significantly enriched among the GO network (**Figs 3 and S3**). The enrichment of CAZymes, redox molecules and nutrient transporters is consistent with enriched functional GO classes that were observed among *pf2ko* differentially expressed genes [13]. We found it striking that genes encoding for TFs were particularly enriched in the high-confidence set of 412 direct targets (**S3 Fig**). They made up 9.7% of these genes in contrast to 3.5% of the total genes annotated for SN15. Five TFs were directly targeted as well as positively regulated, presenting a possible indirect mechanism by which PnPf2 coordinates gene expression (**S1 Table**).

### 2.4. PnPf2 is the predominant transcriptional regulator of host-specific virulence

The identification of TFs as prominent PnPf2 targets suggested they could be key components with an intermediate role in controlling virulence. This prompted a functional exploration to expand the regulatory knowledge for PnPf2. Three TF genes, that were direct targets and are significantly downregulated in *pf2ko* [13], were targeted for deletion (**S1A Table**). These included *SNOG_03490* (*PnPro1*), *SNOG_04486* (*PnAda1*) and *SNOG_08237*. The identification of two distinct PnPf2 ChIP-seq motifs containing CGG triplets (**Fig 2**) also suggested additional Zn_2_Cys_6_ TF involvement in DNA binding and virulence co-regulation. Therefore, we simultaneously targeted the putative PnPf2 paralogue *SNOG_08565* (25.4% sequence identity) [15] and *SNOG_03067* (*PnEbr1*), which is co-expressed with *PnPf2*, *ToxA*, *Tox1* and *Tox3* high during early infection (**S4 Fig**) and has virulence regulating orthologues (summarised in **S1B Table**).

**Fig 4:**
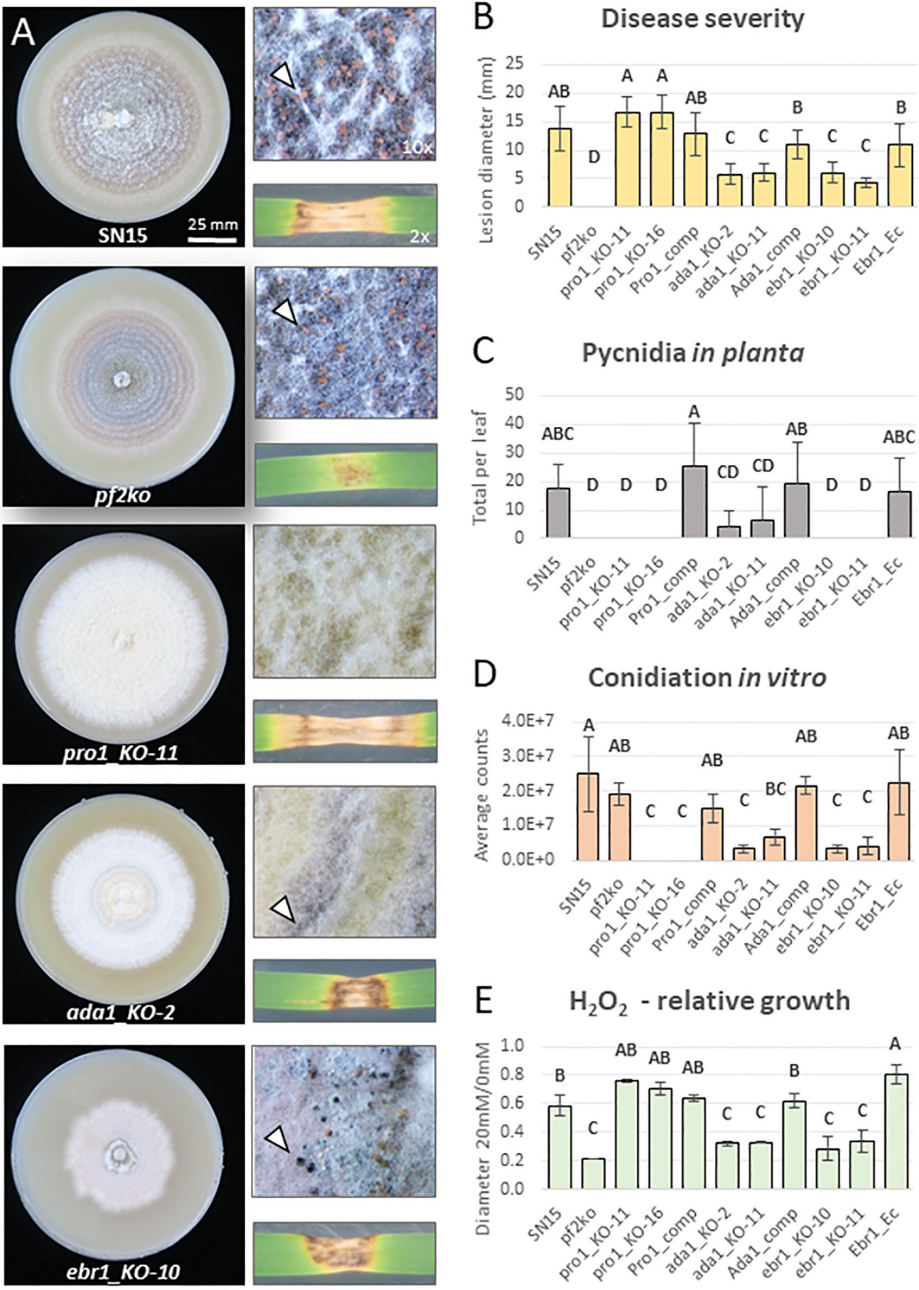
Phenotypic assessment of transcription factor (TF) gene deletion mutants relative to SN15 and *pf2ko*. **A)** Representative images after 12 days of growth on nutrient-rich agar (V8PDA) and infection on detached wheat leaves (cv. Halberd). Arrows demonstrate pycnidia if they were detected in the respective mutants. **B)** Average lesion sizes (replicates = 10) representing disease severity. **C)** Average Pycnidia counts (replicates = 10), a measure of pathogenic fitness following the infection. **D)** Average conidial (pycnidiospore) counts on V8PDA (replicates = 3). **E)** Growth inhibition on 20mM H_2_O_2_ relative to 0mM on minimal medium agar (replicates = 3). Error bars indicate standard deviations and letters indicate statistically distinct groupings by ANOVA with Tukey’s-HSD (P<0.05).

Gene deletion strains for the five TFs were phenotypically characterised in comparison to wildtype SN15 and *pf2ko*. The *pro1_KO*, *ada1_KO* and *ebr1_KO* deletion mutants presented distinct phenotypic abnormalities (**Fig 4**). The *pro1_KO* mutants were abolished in their ability to form pycnidia and sporulate both during infection and on nutrient-rich agar. However, vegetative growth was expansive in both conditions (**Fig 4A and 4B**), suggesting PnPro1 acts to suppress hyphal development. Although *PnPro1* is positively-regulated by PnPf2, there was no distinct phenotypic overlap with the *pf2ko* mutant. The *ada1_KO* mutant was significantly reduced in virulence (**Fig 4A and 4B**). Dark brown discolouration at the site of infection suggested a hypersensitive response had contained the infection. We also observed an increased susceptibility to oxidative (H_2_O_2_) stress for *ada1_KO* mutants similar to *pf2ko*. Furthermore, sporulation was reduced in *ada1_KO* relative to SN15 (**Fig 4C–4E**). The *ebr1_KO* mutants exhibited vegetative growth defects with an uneven growth perimeter around the colony edges coincident with perturbed virulence (**Fig 4A and 4B**). Similar hyphal-branching defects were described following deletion of *PnEbr1* orthologues in *Fusarium* spp. [33,34]. Interestingly, the *ebr1_KO* mutants were also susceptible to H_2_O_2_ stress at a level comparable to *pf2ko* and *ada1_KO*. Furthermore, pycnidia were abnormally developed, although still viable for the production of pycnidiospores, but were not detected on infected leaves (**Fig 4C and 4D**). We did not observe morphological or virulence defects for the *08237_KO* or *08565_KO* mutants (**S2 Text**) and the necrosis-inducing activity for fungal culture filtrate on several wheat lines differentially sensitive to effectors (**S3 Text**) did not change for any of the novel TF mutants investigated.

**Fig 5:**
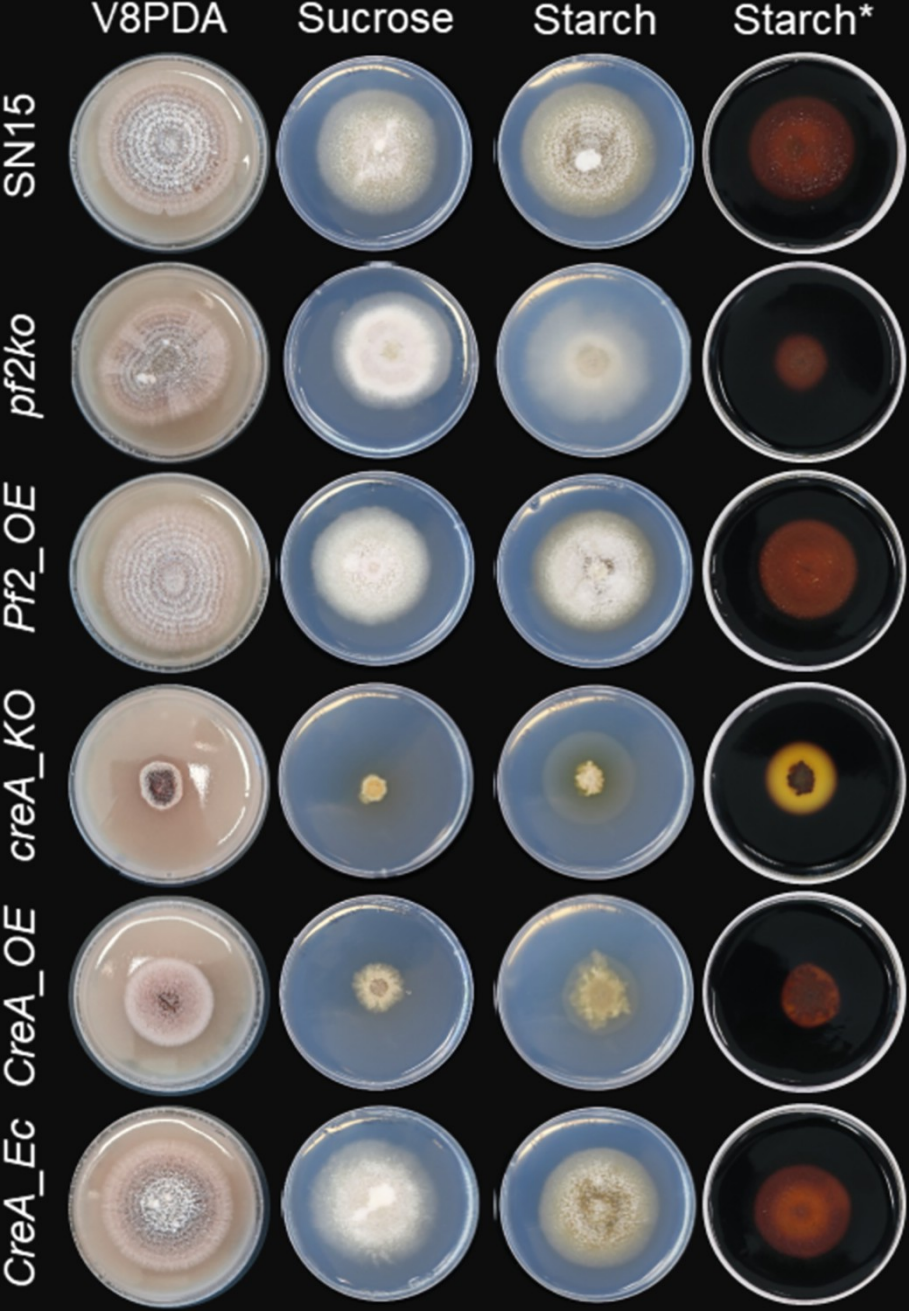
Assessment of *PnPf2* and *PnCreA* mutant growth on different substrates. Images following 12 days of growth on nutrient-rich V8PDA and minimal-medium agar with a primary (sucrose) or secondary (starch) carbon source. Wildtype SN15 and the respective knockout (*KO*), overexpression (*OE*) or ectopic-integrated control (*Ec*) mutants are listed on each row. *Starch plates were post-stained with Lugol’s iodine to assist visualisation of starch hydrolysis (clear halo) which was enhanced in the *creA_KO* mutant despite growth defects and moderately reduced in the *pf2ko* mutant. Quantification of growth speed and further phenotypic analysis is provided (**S2 Text**).

During the course of this study the carbon-catabolite repressor (CCR) element was modelled as the binding site for the Cre-1 TF that suppresses CAZyme expression in *N*. *crassa* [22]. We noted this was nearly identical to a motif (5‘-RTSYGGGGWA-3’) that is also enriched in PnPf2-regulated gene promoters [13] but not identified from the ChIP-seq peaks. Since Cre-1 orthologues are conserved CCR regulators in filamentous fungi [35,36], and since the CCR element is also enriched in PnPf2 regulated gene promoters, a putative Cre-1 orthologue (PnCreA) was investigated in *P*. *nodorum*. Both *PnCreA* overexpression and gene-deletion mutants (*CreA_OE* and *creA_KO*) were created and then investigated alongside *pf2ko* and a *PnPf2* overexpression mutant (*Pf2_OE*). Despite clear phenotypic-growth abnormalities (**Fig 5**), neither the *CreA_OE* nor *creA_KO* mutants exhibited virulence defects on wheat leaves (**S2 Text**) or changes in culture filtrate necrosis-inducing activity. The *creA_KO* strain was enhanced in starch utilisation (**Fig 5**), an indicator substrate for CCR activity [37]. In contrast, there was a moderate reduction of *pf2ko* to utilise starch, similar to observations in other fungal *PnPf2*-orthologue mutants [17,19,21]. These results support contrasting roles between PnCreA and PnPf2 for the regulation of some CAZyme-related genes but these do not appear to be significant factors during infection.

## 3. Discussion

Prior to this research, PnPf2 had been identified as an important regulator of *P*. *nodorum* virulence on wheat [12,13], but details and mechanistic insights were missing. We sought to take further steps and establish the DNA-binding elements targeted by PnPf2 and identify genes that were directly regulated. Two distinct regulatory motifs M1 and M2 were identified and linked to positive gene-regulation by PnPf2. M1 was strikingly similar to an enriched sequence in AbPf2 positively-regulated gene promoters [11], possibly representing a conserved Pf2 binding mechanism. It will be pertinent to explore this motif as a regulatory target for other fungal Pf2 orthologues [11–14,16–21]. Interestingly, the M2 motif matches the extensively characterised AmyR regulatory-response element in *A*. *nidulans* [38,31]. Polysaccharide metabolism has long been established as a regulatory function for AmyR [39,40]. Therefore, some shared regulatory pathways likely exist with Pf2 orthologues given the evidence for at least one conserved binding mechanism. However, there are major a.a polymorphisms between AmyR and Pf2 orthologues at the Zn_2_Cys_6_ DNA-binding domain [15] and M1 has not been reported as an AmyR target despite extensive motif investigation [38,31]. It is therefore conceivable that M1 is a regulatory binding element unique to Pf2 orthologues and therefore useful to identify putative direct targets such as *ToxA* in *P*. *nodorum*. The yeast-one-hybrid analysis conducted here suggested an endogenous yeast TF(s) also shares affinity for M1 and M2 (**S1 Fig**). This may be explained by the abundance of Zn_2_Cys_6_ family TFs encoded in fungal genomes which similarly target CGG-containing motifs [15,26,41]. However, when using the M1^x2^ and M2^x2^ motif configurations which did not fully auto-activate in yeast, a PnPf2 interaction was not observed. This further indicated coregulatory factors absent in the heterologous yeast system are required for PnPf2 promoter binding *in situ*.

The ChIP-seq PnPf2-DNA binding dataset facilitated the identification of *P*. *nodorum* genes under direct PnPf2 regulation. Among these genes are the *Tox3* effector and the adjacent gene, *SNOG_08982*, encoding a protein disulphide isomerase. This class of protein catalyses cysteine-cysteine bond formation which has been connected to fungal effector protein production [42]. We are currently investigating the involvement of *SNOG_08982* in the post-translational modification of Tox3 and other effectors. PnPf2 binding was also detected in the *Tox1* promoter. A partial reduction in *Tox1* expression was reported in the *pf2ko* mutant [13], indicating that PnPf2 is not essential but enhances expression under favourable conditions. *ToxA* is only expressed *in planta*, but is PnPf2 dependent [12]. Despite multiple instances matching the M1 motif, there was little evidence for PnPf2-*ToxA* promoter binding, suggesting chromatin inaccessibility or the absence of essential binding-cofactors under the ChIP culture conditions. Direct PnPf2 regulation of *Tox267* was not evident. The other recently-cloned effector gene *Tox5* is not present in the SN15 isolate used in this study but is homologous to *Tox3*, and may be under PnPf2 control [6]. Nevertheless, several other effector-like genes were identified as direct PnPf2-regulated targets (**Table 2**). Importantly this analysis provided strong evidence that PnPf2 is a key direct-regulator of effectors, the major *P*. *nodorum* virulence factors in the lifestyle of this pathogen.

Evidence for regulation of effector expression has been reported for another *P*. *nodorum* TF PnCon7 [43], yet its apparent requirement for fungal viability renders it difficult to investigate a precise functional role. Here, the enrichment of TFs as direct PnPf2 targets (**Fig 3**) indicated other TFs act as intermediates in controlling NE expression and virulence (**S1A Table**). This prompted their functional investigation in an effort to expand the regulatory knowledge related to PnPf2. We did not observe any change in the necrosis-inducing activity on wheat of fungal culture filtrates derived from the respective mutants, indicating these TFs are not required for NE production. However, developmental virulence roles, including oxidative stress tolerance and hyphal development, was identified for *P*. *nodorum* PnAda1. It is possible that the direct regulation of *PnAda1* by PnPf2 is required for resistance to oxidative stress and plays a role during infection, as susceptibility was also observed for the *pf2ko* mutant. This provides an opportunity to identify genes that are directly regulated by PnAda1 and reveal the shared regulatory targets with PnPf2. The PnCreA orthologue of *N*. *crassa* Cre-1 was also investigated, following the striking observation that the *N*. *crassa* Cre-1 CCR element (5′-TSYGGGG-3’) was enriched in PnPf2-regulated gene promoters [13]. Furthermore, Cre-1 and the PnPf2 orthologue Col-26 are both key components of a transcriptional network controlling CAZyme production in *N*. *crassa* [22–24,44]. Here, the *creA_KO* strain displayed an enhanced capacity to utilise starch, which was moderately impaired in the *pf2ko* mutant (**Fig 5**). This indicates PnCreA and PnPf2 shared a similar function to the respective *N*. *crassa* orthologues [21]. Despite vegetative growth abnormalities on agar, there was no distinct change in the virulence profile of either the *creA_KO* or *CreA_OE* mutants (**S2 Text**). We also failed to detect the CCR element in the promoters of *ToxA*, *Tox1*, *Tox3* or *Tox267* (**S1 File**). This suggests that the regulation of host-specific virulence factors critical for *P*. *nodorum* infection is not subject to CCR by PnCreA.

**Fig 6:**
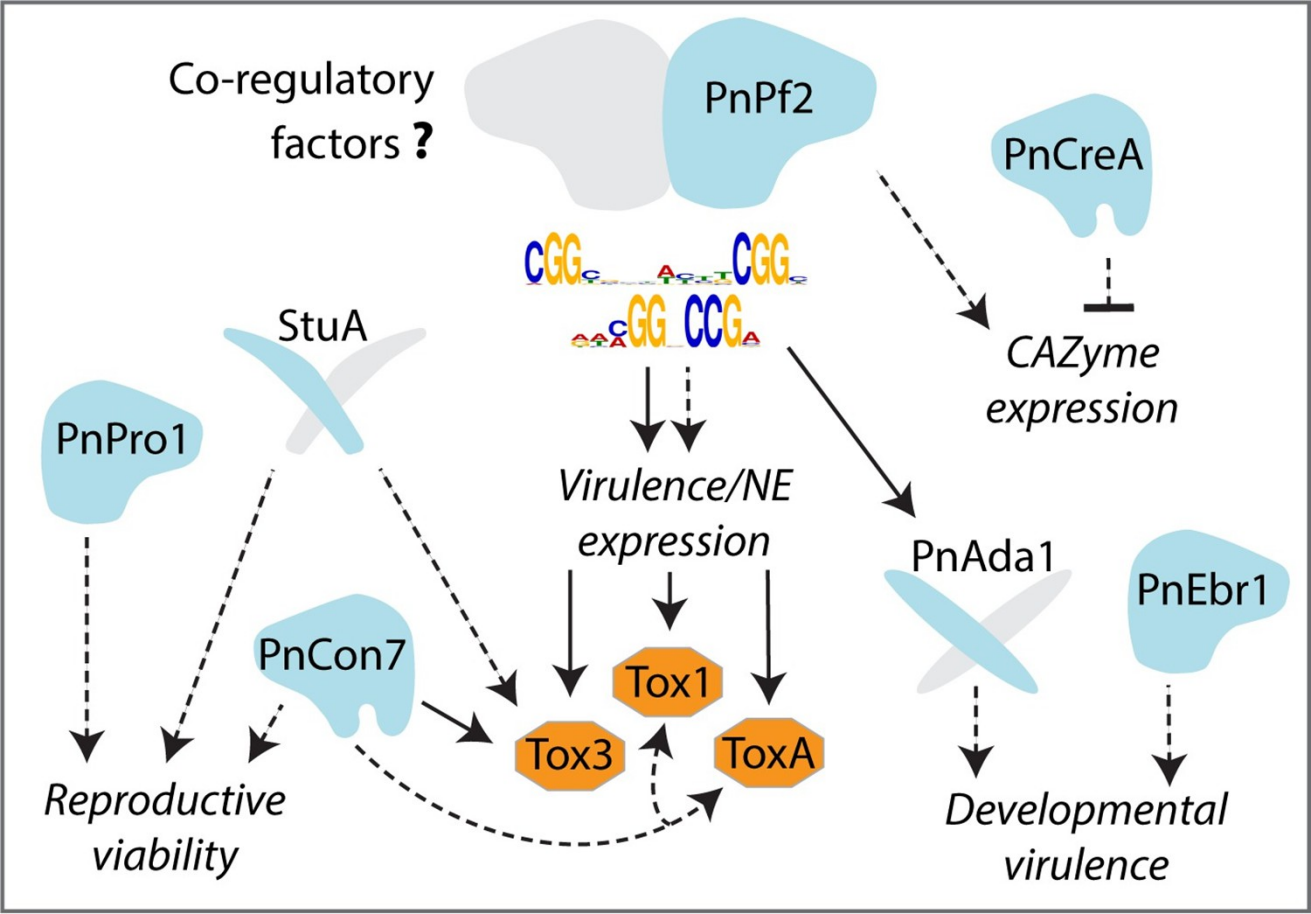
The proposed model of PnPf2 in the virulence of *P*. *nodorum* based on the findings presented in this study relative to characterised transcription factor (TF–blue shapes) and necrotrophic effector (NEs–orange shapes) regulatory pathways. Grey shapes depict putative interacting proteins, dashed arrows depict gene regulation and solid arrows direct regulation. The effector *Tox3* is directly regulated by PnPf2 and *ToxA*, based on promoter-motif and gene expression data, is likely a direct target during plant infection. PnPf2 also directly targets the *Tox1* promoter and quantitatively regulates expression. CAZymes are also regulated by PnPf2, with a subset putatively repressed by PnCreA for which no distinct role in virulence has been established. Developmental virulence, such as oxidative stress tolerance and hyphal growth, were processes attributed in this study to the PnPf2 target PnAda1 and the co-expressed TF PnEbr1. PnPro1 is essential for reproduction by sporulation, as is StuA based on a previous investigation [45]. Elsewhere, PnCon7 has been reported to regulate effector expression but is an essential viability factor [43].

This investigation, along with all previous studies investigating TFs in *P*. *nodorum* [45,46,12,43], indicate that PnPf2 is the predominant characterised regulator directly and specifically coordinating virulence. Our model is proposed (**Fig 6**). Having expanded our understanding, it also raised some key questions. For many genes directly targeted by PnPf2, differential expression in *pf2ko* has not been observed *in vitro* or *in planta* (306 of 412 high-confidence targets). Such discrepancies are frequently reported in ChIP-seq experiments on filamentous fungi (**S2 Table**). One aspect to consider is that functional TF binding requires specific cofactors/co-regulators before gene expression is eventually modulated [47,48]. Furthermore, TF-DNA interactions can be redundant or non-functional [49–51]. It is therefore plausible that many binding sites are transiently occupied by PnPf2 in this manner, acting as a biological sink. A change in the epigenetic landscape, for example during growth *in planta*, could open up genomic regions for which PnPf2 exhibits a high affinity and then can freely bind. Performing PnPf2 ChIP during early infection will likely prove highly useful in this regard if sufficient fungal material can be obtained. ChIP-seq targeting histone marks specific for euchromatin or heterochromatin under infection conditions, or methylation-sensitive sequencing are alternatives to provide insight into the genome accessibility of PnPf2 [14,52–54]. The identification of both the M1 and M2 motifs carrying alternatively oriented ‘CGG’ triplets, typical of Zn_2_Cys_6_ monomers [26], was suggestive that PnPf2 dimerises with at least one other Zn_2_Cys_6_ TF. We did not identify any co-regulatory role in NE production for the putative paralogue *SNOG_08565* or the co-expressed Zn_2_Cys_6_ TF *PnEbr1*. Therefore, to expand our current model (**Fig 6**) and construct an effector/virulence regulatory network in *P*. *nodorum*, future investigations will seek to identify any potential co-regulators, for example through co-immunoprecipitation/affinity purification analysis or a yeast-two-hybrid screens, to delineate the PnPf2-DNA binding mechanisms. These will be undertaken alongside functional investigation of individual domains such as the PnPf2 ‘middle homology region’ and C-terminal disordered region, to provide insight into the upstream signalling pathways that activate or repress PnPf2 activity.

To conclude, this study presents direct evidence of DNA binding in a Pf2 orthologue, where virulence-regulatory functions are consistently observed in phytopathogenic fungi. In *P*. *nodorum*, PnPf2 remains the predominant transcriptional regulator of host-specific virulence characterised and directly controls effector expression. The current research on PnPf2 now provides a platform to further investigate its signalling pathways and molecular interactions that could be inhibited for targeted disease control.

## 4. Materials and methods

### 4.1. Phylogeny and PnPf2 domain analysis

The *P*. *nodorum* annotated genome for the reference isolate SN15 [55] was used consistent with the previous RNA-seq analysis [13]. The PnPf2 polypeptide sequence was submitted to Interproscan (Release 82.0) to identify conserved domains [56]. NLStradamus was used to predict the nuclear localisation signal [57]. The disordered region was predicted using IUPRED2A [28]. Alignment of orthologues identified previously [15] focusing on those characterised in the scientific literature was undertaken using MUSCLE and a maximum-likelihood tree built using the LG distance model and 100 bootstraps with PhyML [58,59].

### 4.2. Generation and assessment of fungal mutants

The molecular cloning stages, primers used, the constructs generated and a summary/diagrammatic overview of the *P*. *nodorum* mutants generated in this study are detailed in **S3 Text**, which also includes procedures relevant to mutant cultivation, phenotypic characterisation and gene-expression analysis.

### 4.3. Chromatin immunoprecipitation sample preparation

The *Pf2-HA*, *Pf2-HA_OE* and *pf2-HA_KO* strains were prepared following 3 days standardised growth in 100 mL Fries3 liquid medium (**S3 Text**). Prior to harvesting, a 5 mL crosslinking solution (10% w/v formaldehyde, 20 mM EDTA and 2 mM PMSF dissolved in 50 mM NaOH) was added with continuous shaking at 100 rpm for 10 min. To this, 5 mL quenching solution (1.25 M glycine) was added before another 10 min shaking. Whole protein extracts were then obtained as described (**S3 Text**) with modifications for ChIP. The 50 mM Tris was replaced with 50 mM HEPES in the lysis buffer while gentle rotation of the resuspended fungal material was replaced by eight rounds of sonication using a Bandelin (Berlin, Germany) UW3100+SH70+MS73 tip sonicator to fragment the fungal DNA (set at 15 sec on/off with 60% amp and 0.8 duty cycle). Samples were held in an ice block during sonication. The supernatant was then retrieved from two rounds of centrifugation (5000 g, 4°C for 5 min). A 100 μL aliquot of the supernatant was reserved as an ‘input control’ against which ChIP samples were to be normalised. A 1000 μL aliquot was then precleared for immunoprecipitation by gently rotating with 20 μL Protein A dynabeads (10001D - Thermofisher, Waltham, Massachusetts) for 1 hr at 4°C. The supernatant was then retrieved and incubated with 2.5 μg anti-HA polyclonal antibody (71–5500—Thermofisher) for 16 hrs at 4°C. Another 20 μL Protein A dynabeads were then added and gently rotated for 2 hrs at 4°C. The dynabeads were then retrieved and washed twice with 1 mL ice-cold lysis buffer, once with high-salt buffer (lysis buffer + 500 mM NaCl), once with LiCl buffer (250 mM LiCl, 10 mM Tris-HCl, 1 mM EDTA, 0.5% NP40 and 0.5% NaDOC) and once with TE buffer (10 mM Tris-HCl, 1 mM EDTA, pH 8). Samples were then incubated in a shaking incubator for 10 min (300 rpm, 65°C) with 200 μL elution buffer (0.1 M NaHCO_3_, 10 mM EDTA and 1% SDS) before transferring the supernatant to a fresh tube. The input control was also supplemented with 100 μL elution buffer at this stage and 8 μL NaCl solution (5 M) was added to both samples before de-crosslinking for 16 hrs at 65°C. To these samples, 200 μL of H_2_O and 100 μg RNAse A (QIAGEN, Hilden, Germany) were added before incubating for 1 hr at 65°C. Ten μg Proteinase K (Sigma-Aldrich, St. Louis, Missouri) was then added before incubating a further 1 hr at 50°C.

For ChIP-qPCR, DNA (for both the *Pf2-HA*, *Pf2-HA_OE* and *pf2-HA_KO* ChIP and input control samples) was recovered from Proteinase K treated samples using the GenElute PCR purification kit (Sigma-Aldrich). For ChIP-seq, DNA was purified from the Proteinase K treated samples by mixing in 1 volume (400 μL) of phenol:chloroform, centrifuged for 5 min at 16000 g and the aqueous phase retrieved. To this, 400 μL chloroform was added, mixed and spun (16000 g 5 min) before 350 μL of the aqueous phase was transferred to a fresh tube. 35 μL sodium acetate (3 M, pH 5.2) was added with 1 μL of glycogen (20 mg/mL). Samples were mixed by inversion and 1 mL 100% ethanol added before precipitation at -80°C for 1–2 hrs. Pellets were retrieved by spinning 16000 g for 10 min at 4°C, washed in 1 ml of ice-cold 70% ethanol, dried and resuspended in 30 μL Tris-Cl (10 mM). An additional biological replicate (r2) of ChIP-seq DNA was prepared following the MAGnify Chromatin Immunoprecipitation System (Thermofisher) using the same mycelial crosslinking/sonication procedure described above to prepare the chromatin.

The DNA for all samples was measured using a Tapestation system (Agilent, Santa Clara, California). For replicate #1 samples, 10 ng was processed using the TruSeq ChIP Library Preparation Kit (Illumina, San Diego, California). Libraries were size-selected (100–300 bp) and split across four separate lanes for sequencing in a NextSeq 500 sequencer (Illumina) to obtain 2 x 75 bp paired-end reads. For the replicate DNA samples (r2), libraries were prepared using the xGen cfDNA & FFPE DNA Library Preparation Kit (Coralville, Iowa), size selected (100–500 bp) and sequenced using a NovaSeq 6000_SP sequencer (Illumina). All library preparations and sequencing were undertaken by the Australian Genome Research Facility (Melbourne, Australia).

### 4.4. ChIP-seq analysis

An overview of the data analysis pipeline including QC of raw reads, genome mapping, ChIP-seq peak/summit calling and filtering by replicate/control samples, target gene prediction, ChIP-qPCR validation, GO enrichment analysis and motif position-weight-matrix (PWM) modelling is provided (**S1 Text**).

#### 4.4.1. Raw read filtering, mapping and peak/summit calling

Raw reads were checked using FASTQC (Version 0.11.9) [60] and the adapter sequences were trimmed using Cutadapt (Version 1.15) along with nucleotides where the Illumina quality scores were below 30 [61]. Optical duplicates were then removed using the ‘dedupe’ option in Clumpify (version 1.15) from the BBTools package [62]. Reads were subsequently mapped to the SN15 genome [55] using BWA-MEM [63]. Reads mapping to a single locus as the best match (primary alignments) were retained for downstream analysis using SAMtools [64]. MACS (Version 3.0.0) was used for calling enriched peaks and summits from ChIP sample reads relative to the input samples. A Q-value peak enrichment threshold of 0.01 was used and the BAMPE option utilised to assess read depth from cognate pairs [65,66]. One biological replicate (r1) for *Pf2-HA* and *Pf2-HA_OE* was retained as the primary dataset. Peaks/summits were retained only if the they were also detected in replicate r2 using MAnorm (maximum summit distance 100 bp, window size 400 bp) [67] and did not overlap peaks called from the negative control dataset *pf2-HA_KO*.

#### 4.4.2. Modelling binding-site motifs

The overlapping peak regions identified from the *Pf2-HA* and *Pf2-HA_OE* samples were merged using MAnorm [67] to create a consensus set harbouring putative PnPf2 binding sites. From this set, overrepresented PWMs up to 20 bp long were modelled with MEME (version 5.5.5) [68,69]. For the resulting PWMs, 500 bp genomic regions centred at ChIP-seq summits were extracted and analysed using CentriMo (Version 5.5.5) to obtain motif proximity relative to summits for both the *Pf2-HA* and *Pf2-HA_OE* datasets [70]. Gene promoters (spanning annotated transcription start sites to the nearest upstream gene feature or 1500 bp) with ≥ 1 occurrence of each motif were determined using FIMO [71]. These were cross-referenced with the differentially expressed genes (i.e. expressed significantly up or down in *pf2ko* relative to SN15) defined in a previous RNA-seq analysis [13]. Fisher’s exact test with Bonferroni corrected P-values [72] was used to identify these gene-promoter sets significantly enriched (P_adj_ < 0.01) for the respective motifs vs the background rate in SN15.

#### 4.4.3. PnPf2 target gene-promoter analysis

Genes targeted by PnPf2 were determined based on the proximity of summits to annotated genes using ChIPseeker (Version 1.38.0) [73]. Genes with ≥1 promoter summit from the *Pf2-HA* or *Pf2-HA_OE* datasets were considered PnPf2 targets. High-confidence PnPf2 targets had a promoter summit in *Pf2-HA* and *Pf2-HA_OE*. ChIP-qPCR was then undertaken to verify ChIP-seq peak enrichment. Quantitative PCR primer pairs (**S3 Text**) were designed to flank ChIP-seq summits in a selection of gene promoters (*ToxA*, *Tox1*, *Tox3*, *SNOG_03901*, *SNOG_04486*, *SNOG_12958*, *SNOG_15417*, *SNOG_15429*, *SNOG_16438*, *SNOG_20100* and *SNOG_30077*) and non-summit control regions (*Act1* and *SNOG_15429* coding sequences and the *TrpC* terminator). The ‘input %’ values were calculated for each sample using the method described previously [74] and used to calculate fold-differences (normalised to *Act1*) for *Pf2-HA* and *Pf2-HA_OE* relative to the *pf2-HA_KO* control. Pearson’s correlation coefficient was calculated for the ChIP-qPCR fold-difference vs ChIP-seq -Log_10_(Q-values) at the respective loci and used as the test statistic to assess whether the association was significant (SPSS version 27.0).

The PnPf2 target genes were cross-referenced with the *pf2ko* expression patterns (expressed significantly up or down in *pf2ko*) defined previously [13] to link direct binding with the modulation of gene expression. The most recent SN15 effector-like gene annotations [32] were compiled among the PnPf2 targets and putative homologues identified from corresponding UniProt records (release 2024_01) [75]. Both the high-confidence and total PnPf2 target-gene sets were used to identify the overrepresented (P < 0.01) GO terms documented previously [13] using the ‘enricher’ function in the Clusterprofiler package (Version 4.10.0) [76].

#### 4.4.4. Testing PnPf2-motif interactions

Yeast-one-hybrid experiments, as well as p53 control reactions, were carried out as described in the Matchmaker Gold Yeast One-Hybrid Library Screening System User Manual (Clontech Laboratories). All transformations were performed as per protocol described in the Yeastmaker Yeast Transformation System 2 user manual (Clontech Laboratories). Briefly, single, double and triple tandem repeats of either the M1 (5’-ATAGGCCCGA-3’) or M2 motif (5’-CGGTCGTATTTCGGT-3’) were cloned into yeast-integrative vector pAbAi as *KpnI*/*Hind*III fragments (M1^x3^ and M2^X3^ only), or via PCR and subsequent T4 Polynucleotide Kinase and T4 Ligase ligation as per manufacturer’s instructions (NEB). Confirmation of the motifs’ presence adjacent to the AUR1-C gene were confirmed by PCR and Sanger sequencing. These pAbAi-motif vectors were linearised by PCR before being transformed into *Saccharomyces cerevisiae* Y1HGold strain and subsequently plated on synthetic drop-out defined (SD) media lacking uracil (-URA). The PnPf2 DNA sequence was cloned into the constitutive vector pGADT7 using Gibson assembly. pGAD-PnPf2 and pGADT7 were transformed into Y1HGold carrying the integrated motifs, and all recovery transformation cultures standardised to the same relative optical-density 600 nm values. Twenty μl of each transformant were spot plated in 10-fold serial dilution in 0.9% *w/v* NaCl on selective SD media lacking leucine (-LEU) with and without Aureobasidin A (AbA; MedChemExpress) at 500 ng/μL final concentration.

## Supporting information

S1 Text Chromatin immunoprecipitation strain quality-control assessment and overview of the ChIP-seq pipeline and datasets obtained. (DOCX)

S2 Text Supplemental transcription factor mutant phenotype assessment. (DOCX)

S3 Text Supplemental materials and methods. Includes a description of all the strains generated and primers used in this study.
(DOCX)

S1 Table Transcription factor (TF) genes relevant to this study. **A)** Positively regulated direct PnPf2 targets. **B)** Rationale for functional investigation of specific TFs.
(DOCX)

S2 Table Congruency between genes detected by TF ChIP-seq and RNA-seq in alternative publications on other filamentous fungi. (DOCX)

S1 Fig Yeast-one-hybrid of PnPf2 against Y1HGold. Yeast cells were carrying motif M1 in single (M1^x1^), double (M1^x2^) or triple (M1^x3^) tandem repeats, or M2 in single (M2^x1^), double (M2^x2^) or triple (M2^x3^) tandem repeats. PnPf2 constitutively expressed from the pGADT7 vector as well as the empty vector (pGADT7) showed growth on SD -LEU media containing Aureobasidin A at 500 ng/μL (AbA_500_) for both the single and triple motif copies of M1 and M2, with or without PnPf2. This indicates activation of the *AUR1-C* reporter gene is due to the presence of the motifs alone, suggesting an endogenous yeast factor(s) can act to bind these DNA motifs in the Y1HGold background, independent of PnPf2. The dual-tandem copies of M1 and M2 were not auto-activated, but no increased activation of the *AUR1-C* reporter was observed in the presence of PnPf2.
(PDF)

S2 Fig A depiction of the PnPf2 targeting of characterised effector genes in *P*. *nodorum* SN15. The *Pf2-HA* and *Pf2-HA_OE* ChIP-seq read peaks are presented at the *Tox3*, *Tox1*, *ToxA* and *Tox267* promoters. Peak summits were evident in the *Tox3* and *Tox1* promoters. Red dots represent instances of the M1 motif (5’-RWMGGVCCGA-3’) and blue dots M2 (5’-CGGCSBYWYBKCGGC-3’).
(PDF)

S3 Fig Original GO-enrichment analysis presented in **Fig 3** including both **A)** the high-confidence PnPf2-targeted genes (total = 412) and **B)** all candidate PnPf2 targets (total = 1253).
(PDF)

S4 Fig A heatmap depiction of *Parastagonospora nodorum* SN15 hierarchical cluster analysis. Clustering was based on microarray gene-expression data during infection (*in planta*) or axenic (*in vitro*) growth obtained from a previous study [77]. Genes were divided into the 10 most distant clusters to identify genes co-expressed with *PnPf2*, *ToxA*, *Tox1* and *Tox3*, which included the Zn_2_Cys_6_ transcription factor *PnEbr1* (*SNOG_03037*) therefore investigated in this study.
(PDF)

S1 File PnPf2 ChIP-seq regulation data. **Tab 1)** A spreadsheet detailing the genomic coordinates for ChIP-seq peak regions [columns A-C], the respective summit loci [D], the summit -Log_10_(Q-values) measuring the relative difference of ChIP reads to input controls [E], the nearest adjacent gene transcription start sites, strand and the respective distances [F-H]. Data for the *Pf2-HA* strain span columns A-H and the *Pf2-HA_OE* strain column I-P. Genomic coordinates of merged peaks from both datasets using MAnorm [67] are shown in columns Q-T. **Tab 2)** A spreadsheet summarising PnPf2 regulation data across the *P*. *nodorum* SN15 genome for the respective annotated genes [column A]. Listed are whether ChIP-seq promoter summits were called from the *Pf2-HA* and *Pf2-HA_OE* samples [B-C], whether the enriched PnPf2 target motifs [D-E] or the putative PnCreA motif [G] were present in the gene promoter regions, and whether the gene was also down/up-regulated in the *pf2ko* mutant (*in vitro* or *in planta*) [G-J]. Also listed are the functional annotations [K-P]; whether the gene was classed as effector-like [K], a TF [L], and the associated GO/Interpro domain information [M-P]. The final columns list the respective gene expression data for *pf2ko* compared with SN15 either *in vitro* (*iv*) or *in planta* (*ip*) [O-W]. *Information indicated was derived from [13] for comparative purposes. **Predicted effectors derived from [32].
(XLSX)
