# Supplementary material for: Regulatory insight for a Zn2Cys6 transcription factor controlling effector-mediated virulence in a fungal pathogen of wheat
Source: PLoS Pathog. 2024 Sep 23;20(9):e1012536. doi: 10.1371/journal.ppat.1012536 (PMC11419344; doi:10.1371/journal.ppat.1012536)
Supplement: S1 Text — (DOCX) [file ppat.1012536.s001.docx]

**Text S1 – Chromatin immunoprecipitation (ChIP) strain assessment and overview of ChIP-seq/ChIP-qPCR.**

This text provides an assessment of the strains used (**Text S1-Fig. 1**), an overview of the ChIP-seq data generation and analysis pipeline (**Text S1-Fig. 2**) and the ChIP-seq reads generated for peak/summit calling (**Text S1-Table 1**; **Text S1-Fig. 2**).

**
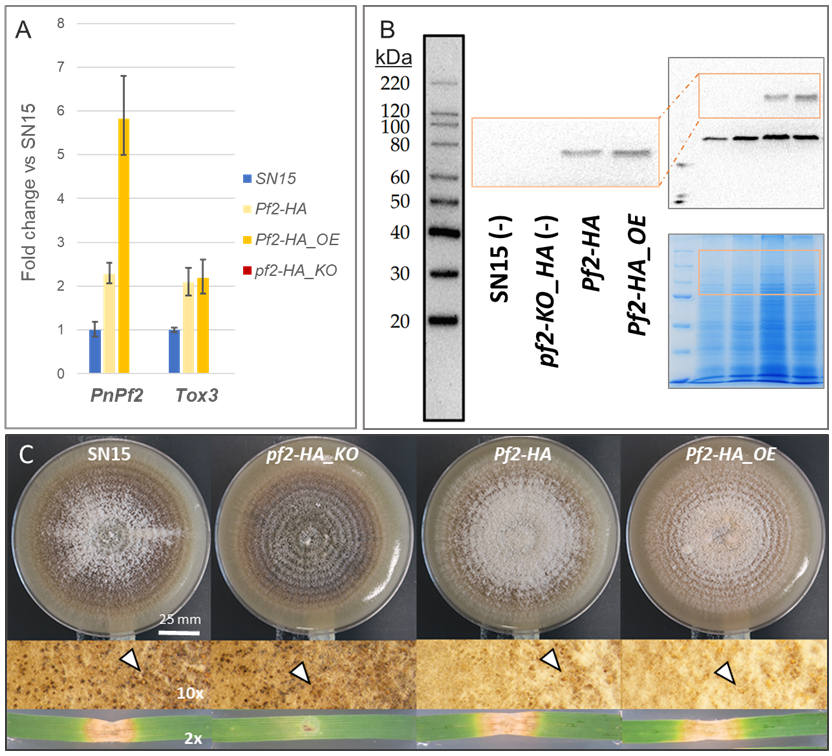
**

**Text S1-Fig.1** Assessment of strains used for ChIP**.
A)** A quantitative PCR analysis that compared the relative gene expression of *PnPf2* and the PnPf2-regulated necrotrophic effector *Tox3* gene. This indicated the 3x haemagglutinin (HA) tag did not inhibit *PnPf2* expression or its activity under the ChIP-seq conditions (Fries3 liquid medium, 72 hrs). Error bars reflect the standard deviation of underlying dCt(*Actin – Target*) values for 3 biological replicates. **B)** A Western blot using an anti-HA antibody on total-protein extracts revealed a band corresponding to the expected PnPf2-HA fusion protein size ~75 kDa under ChIP-seq conditions. The band was not detected in SN15 or the negative control strain *pf2-HA_KO*. The full-size original blot and a corresponding Coomassie Blue stained gel are included for reference. A smaller band was detected in all samples presumed to be non-specific antibody binding. **C)** A phenotypic comparison for the respective mutants relative to the SN15 wildtype. The upper two images represent 12 days growth on V8PDA, with arrows indicating mature pycnidia. The lower images depict representative lesions, 12 days following inoculation on detached wheat leaves (cv. Halberd). This suggested virulence was not inhibited and that PnPf2 was functional in *Pf2-HA* and *Pf2-HA_OE* in contrast to *pf2-HA_KO*.


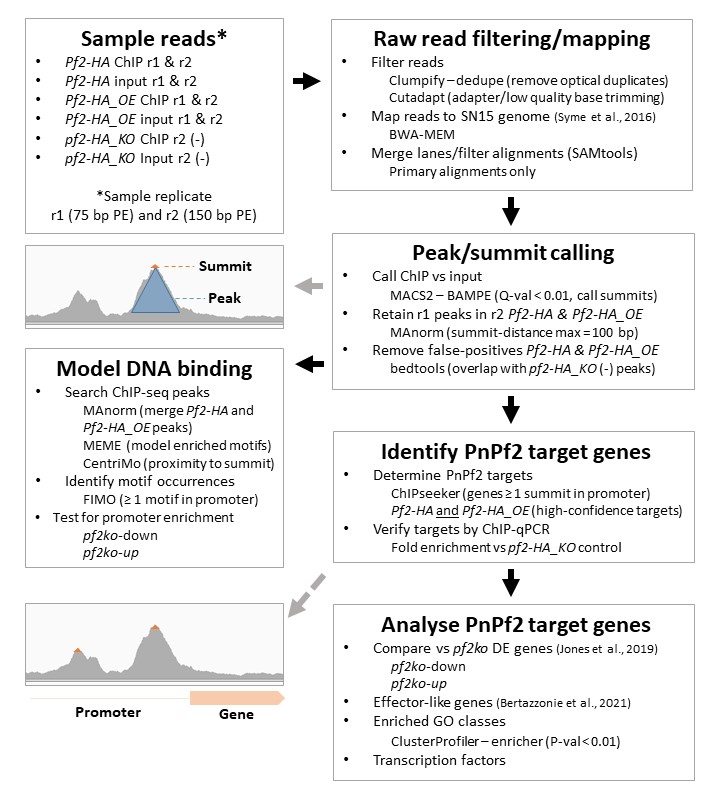


**Text S1-Fig. 2** A diagrammatic overview of the data processing and analysis pipeline followed for ChIP-seq. Grey arrows indicate visual examples of the output from the corresponding stages while black arrows indicate subsequent procedures.

**Text S1-Table 1** ChIP-seq reads mapped to the SN15 genome for calling enriched peaks and summits corresponding to putative PnPf2 binding loci **^A^**

| Strain | DNA | Raw reads | Mapped reads | Sample  summits/peaks | Final  summits/peaks |
| --- | --- | --- | --- | --- | --- |
| *Pf2-HA*  (native) | ChIP r1 | 22,740,293‬ | 12,416,341 | 997 / 740 | 760 / 586 |
|  | Input r1 | 21,762,558 | 13,307,189 |  |  |
|  | ChIP r2 | 50,084,252 | 45,358,150 | 4801 / 3532 |  |
|  | Input r2 | 55,740,410 | 49,303,902 |  |  |
| *Pf2-HA_OE*  (over) | ChIP r1 | 29,524,953 | 17,697,729 | 2213 / 1594 | 2081 / 1536 |
|  | Input r1 | 22,974,742‬ | 15,270,059 |  |  |
|  | ChIP r2 | 57,358,653 | 21,747,502 | 3275 / 2443 |  |
|  | Input r2 | 84,289,290 | 46,843,710 |  |  |
| *pf2-HA_KO*  (-) | ChIP r2 | 40,019,985 | 32,009,296 | 3295 / 2369 | NA |
|  | Input r2 | 36,876,077 | 31,184,282 |  |  |

^A^ The raw reads derived from chromatin-immunoprecipitated (ChIP) or background control (Input) for each sample strain. Reads were mapped as pairs to unique loci after quality control and used to call both enriched peak regions and their (one or more) summits. Final summits/peaks for the *PnPf2-HA* native/overexpression strains (*Pf2-HA*/*Pf2-HA_OE*) were those overlapping in both replicates but not in the negative control (*pf2-HA_KO*).
