# Supplementary material for: Regulatory insight for a Zn2Cys6 transcription factor controlling effector-mediated virulence in a fungal pathogen of wheat
Source: PLoS Pathog. 2024 Sep 23;20(9):e1012536. doi: 10.1371/journal.ppat.1012536 (PMC11419344; doi:10.1371/journal.ppat.1012536)
Supplement: S2 Text — (DOCX) [file ppat.1012536.s002.docx]

**Text S2 – Supplemental transcription factor (TF) mutant phenotypic assessment.**

This text provides observations pertaining to the TF mutants generated in this study not presented in the main text. This includes the deletion mutants targeting the putative SNOG_08237 and SNOG_08565 TFs generated in this study (**Text S2-Fig. 1**) and the *PnCreA* mutants relative to *PnPf2* mutants (**Text S2-Fig. 2**).


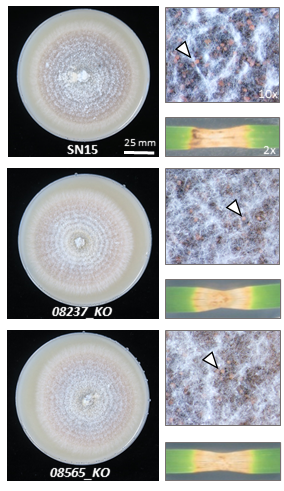


**Text S2-Fig. 1** Assessment of the *08237_KO* and *08565_KO* mutants relative to the SN15 wildtype. Representative images after 12 days of growth on nutrient-rich agar (V8PDA) and infection on detached wheat leaves (cv. Halberd). Arrows demonstrate mature pycnidia detected in the respective mutants.


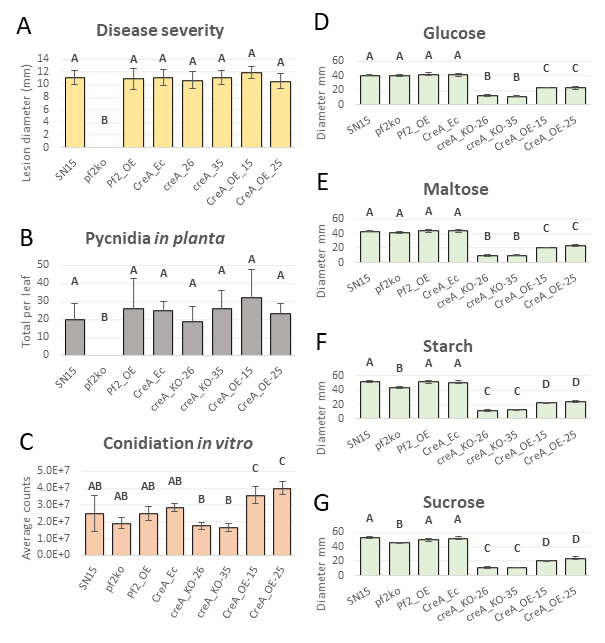


**Text S2-Fig. 2** Phenotypic assessment of *PnCreA* and *PnPf2* knockout (KO), overexpression (OE) or ectopic-integrated control (*Ec*) mutants. **A)** The average lesion sizes (replicates = 10) representing disease severity and **B)** pycnidia counts (replicates = 10), a measure of pathogenic fitness following the infection. **D)** The average conidial (pycnidiospore) counts on V8PDA (replicates = 3) and **D-G)** the colony diameters following 12 days growth on glucose, maltose, starch or sucrose minimal-medium agar (replicates = 3). Error bars indicate standard deviations and letters indicate statistically distinct groupings by ANOVA with Tukey's-HSD (P<0.05).
