# Supplementary material for: Regulatory insight for a Zn2Cys6 transcription factor controlling effector-mediated virulence in a fungal pathogen of wheat
Source: PLoS Pathog. 2024 Sep 23;20(9):e1012536. doi: 10.1371/journal.ppat.1012536 (PMC11419344; doi:10.1371/journal.ppat.1012536)
Supplement: S1 Table — A) Positively regulated direct PnPf2 targets. B) Rationale for functional investigation of specific TFs. (DOCX) [file ppat.1012536.s004.docx]

**Table S1-A** Transcription factor genes predicted as positively regulated direct PnPf2 targets ^A^

| Gene ID | Summit loci | | Motif loci | | Protein length | Protein annotation | Characterised orthologues ^B^ |
| --- | --- | --- | --- | --- | --- | --- | --- |
|  | ***Pf2-HA*** | ***Pf2-HA_OE*** | **RWMGGVCCGA** | **CGGCSBYWYBKCGGC** |  |  |  |
| *PnPro1* (SNOG_03490) | -353; -541 | -270 | - | - | 585 | IPR001138 (Zn2Cys6); IPR021858 (Fun_TF) | AbPro1 (*Ab*), Pro1 (*Cp*), GzZC232 (*Fg*), MoPRO1 (*Mo*), UvPro1 (*Uv*) |
| *PnAda1* (SNOG_04486) | -907 | -903 | -864 | -887 | 654 | IPR004827 (bZIP) | GzbZIP001 (Fg), FpAda1 (Fp), MobZIP10 (Mo) |
| SNOG_08237 | - | -852 | -762 | -853 | 303 | IPR001356 (Homeobox) | CoHox1 (*Co*), MoHox5 (*Mo*), GzHOME004 (*Fg*) |
| SNOG_01243 | -600 | -556 | - | - | 403 | IPR001005 (SANT/Myb) | Myt1 (*Fg*) |
| SNOG_03674 | -382 | -434 | -419 | - | 496 | IPR009071﻿ (HMG box); IPR001660 (SAM) | GzHMG021 (*Fg*) |

^A^ Transcription factors with significantly reduced expression in the *pf2ko* mutant [1] were considered positively regulated. Identified here as PnPf2 direct-targets based on ChIP-seq summit(s) detected upstream of the start codon in their promoter region, whose relative position is provided along with putative PnPf2 target-motif loci.
^B^ Functionally-characterised orthologues in the scientific literature [2] were identified for: *Ab; Alternaria brassicicola*, *Co; Colletotrichum orbiculare, Cp; Cryphonectria parasitica*, *Fg; Fusarium graminearum, Fp; Fusarium pseudograminearum,* *Mo; Magnaporthe oryzae*, *Uv; Ustilaginoidea virens*

**Table S1-B** Rationale for the investigation of novel transcription factors (TFs) in this study

| TF investigated | Involvement | Virulence-associated orthologues ^A^ |
| --- | --- | --- |
| *PnPro1* (SNOG_3490) | Directly-positively regulated by PnPf2 | *AbPro1* (*Ab*), *MoPRO1* (*Mo*), *GzZC232* (*Fg*), *UvPro1* (*Uv*) |
| *PnAda1* (SNOG_04486) | Directly-positively regulated by PnPf2 | *GzbZIP001* (*Fg*), *FpAda1* (*Fp*) |
| SNOG_08237 | Directly-positively regulated by PnPf2 | *CoHox1* (*Co*) |
| SNOG_08565 | Homology to PnPf2 TF [3], possible paralogue | - |
| *PnEbr1* (SNOG_03067) | Co-expressed with *PnPf2*, *ToxA*, *Tox1* and *Tox3* during infection | *EBR1* (*Fg*), *EBR1* (*Fo*), *MoCod2* and *Cnf2* (*Mo*) |
| *PnCreA* (SNOG_13619) | Enriched CreA-binding motif [4] in PnPf2-regulated gene promoters | *CreA* (*Af*), *Cre1* (*Fo*), *CreA* (*Pe*) |

^A^ Putative orthologues were inferred by cross-referencing a previous TF-orthology analysis and literature review [2,3]. Abbreviations: *Ab; Alternaria brassicicola*, *Af; Aspergillus* flavus, Co*; Colletotrichum orbiculare,* *Fg; Fusarium graminearum, Fo; Fusarium oxysporum, Fp; Fusarium pseudograminearum, Mo; Magnaporthe oryzae*, *Pe; Penicillium expansum*.

**References**

1. Jones DAB, John E, Rybak K, Phan HTT, Singh KB, Lin S-Y, et al. A specific fungal transcription factor controls effector gene expression and orchestrates the establishment of the necrotrophic pathogen lifestyle on wheat. Sci Rep. 2019;9: 1–13. doi:10.1038/s41598-019-52444-7

2. John E, Singh KB, Oliver RP, Tan K-C. Transcription factor control of virulence in phytopathogenic fungi. Mol Plant Pathol. 2021;22: 858–881. doi:10.1111/mpp.13056

3. John E, Singh KB, Oliver RP, Tan K-C. Transcription factor lineages in plant-pathogenic fungi, connecting diversity with fungal virulence. Fungal Genetics and Biology. 2021;In press. doi:10.1016/j.fgb.2022.103712

4. Wu VW, Thieme N, Huberman LB, Dietschmann A, Kowbel DJ, Lee J, et al. The regulatory and transcriptional landscape associated with carbon utilization in a filamentous fungus. Proc Natl Acad Sci. 2020;117: 6003–6013. doi:10.1073/pnas.1915611117
