# Supplementary material for: Regulatory insight for a Zn2Cys6 transcription factor controlling effector-mediated virulence in a fungal pathogen of wheat
Source: PLoS Pathog. 2024 Sep 23;20(9):e1012536. doi: 10.1371/journal.ppat.1012536 (PMC11419344; doi:10.1371/journal.ppat.1012536)
Supplement: S2 Table — (DOCX) [file ppat.1012536.s005.docx]

**Table S2** Congruency between genes detected by TF ChIP-seq and RNA-seq in filamentous fungi ^A^

| TF studied ^B^ | ChIP-seq targets | RNA-seq regulated | ChIP-seq + RNA-seq |
| --- | --- | --- | --- |
| PnPf2 (*Pn*) [1] | 412 | 602 | 80 |
| Clr1 (*Nc*) [2] | 164 | 117 | 39 |
| Clr2 (*Nc*) [2] | 84 | 132 | 54 |
| Xlr1 (*Nc*) [2] | 198 | 90 | 23 |
| Tri6 (*Fg*) [3] | 198 | 1614 | 26 |
| FgSR (*Fg*) [4] | 119 | 1790 | Not reported |
| Ros1 (*Um*) [5] | 1913 | 2006 | 790 |
| MoCrz1 (*Mo*) [6] | 346 | 346 (microarray) | 140 |
| CrzA (*Af*) [7] | 102 | 3622 | 50 |
| SrbA (*Af*) [8] | 97 | 987 | 24 |

^A^ Based on the relevant studies in the scientific literature reporting ChIP-seq and RNA-seq differentially-expressed gene datasests. Abbreviations: TF; transcription factor, ChIP; chromatin immunoprecipitation. ^B^ *Af; Aspergillus fumigatus*, *Fg; Fusarium graminearum, Mo; Magnaporthe oryzae*, *Nc; Neurospora crassa,* *Pn; Parastagonospora nodorum*, *Um; Ustilago maydis*.

**References**

1. Jones DAB, John E, Rybak K, Phan HTT, Singh KB, Lin S-Y, et al. A specific fungal transcription factor controls effector gene expression and orchestrates the establishment of the necrotrophic pathogen lifestyle on wheat. Sci Rep. 2019;9: 1–13. doi:10.1038/s41598-019-52444-7

2. Craig JP, Coradetti ST, Starr TL, Glass NL. Direct target network of the *Neurospora crassa* plant cell wall deconstruction regulators CLR-1, CLR-2, and XLR-1. mBio. 2015;6. doi:10.1128/mBio.01452-15

3. Nasmith CG, Walkowiak S, Wang L, Leung WWY, Gong Y, Johnston A, et al. Tri6 is a global transcription regulator in the phytopathogen *Fusarium graminearum*. PLOS Pathogens. 2011;7: e1002266. doi:10.1371/journal.ppat.1002266

4. Liu Z, Jian Y, Chen Y, Kistler HC, He P, Ma Z, et al. A phosphorylated transcription factor regulates sterol biosynthesis in *Fusarium graminearum*. Nat Commun. 2019;10: 1–17. doi:10.1038/s41467-019-09145-6

5. Tollot M, Assmann D, Becker C, Altmüller J, Dutheil JY, Wegner C-E, et al. The WOPR protein Ros1 is a master regulator of sporogenesis and late effector gene expression in the maize pathogen *Ustilago maydis*. PLoS Pathog. 2016;12: e1005697. doi:10.1371/journal.ppat.1005697

6. Kim S, Hu J, Oh Y, Park J, Choi J, Lee Y-H, et al. Combining ChIP-chip and expression profiling to model the MoCRZ1 mediated circuit for Ca2+/calcineurin signaling in the rice blast fungus. PLOS Pathogens. 2010;6: e1000909. doi:10.1371/journal.ppat.1000909

7. Castro PA de, Chen C, Almeida RSC de, Freitas FZ, Bertolini MC, Morais ER, et al. ChIP-seq reveals a role for CrzA in the *Aspergillus fumigatus* high-osmolarity glycerol response (HOG) signalling pathway. Molecular Microbiology. 2014;94: 655–674. doi:https://doi.org/10.1111/mmi.12785

8. Chung D, Barker BM, Carey CC, Merriman B, Werner ER, Lechner BE, et al. ChIP-seq and *in vivo* transcriptome analyses of the *Aspergillus fumigatus* SREBP SrbA reveals a new regulator of the fungal hypoxia response and virulence. PLoS Pathog. 2014;10. doi:10.1371/journal.ppat.1004487
