# Supplementary figures and images for: Regulatory insight for a Zn2Cys6 transcription factor controlling effector-mediated virulence in a fungal pathogen of wheat
Source: PLoS Pathog. 2024 Sep 23;20(9):e1012536. doi: 10.1371/journal.ppat.1012536 (PMC11419344; doi:10.1371/journal.ppat.1012536)

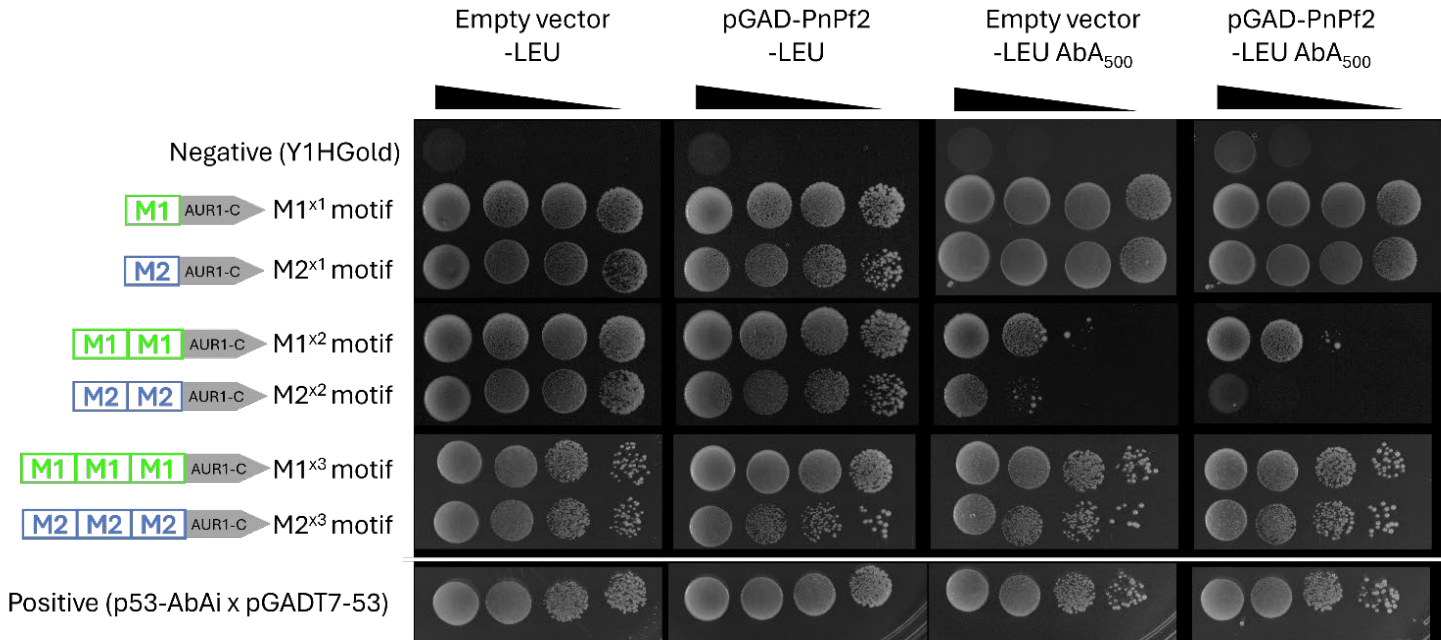

Supplement: S1 Fig — Yeast cells were carrying motif M1 in single (M1x1), double (M1x2) or triple (M1x3) tandem repeats, or M2 in single (M2x1), double (M2x2) or triple (M2x3) tandem repeats. PnPf2 constitutively expressed from the pGADT7 vector as well as the empty vector (pGADT7) showed growth on SD -LEU media containing Aureobasidin A at 500 ng/μL (AbA500) for both the single and triple motif copies of M1 and M2, with or without PnPf2. This indicates activation of the AUR1-C reporter gene is due to the presence of the motifs alone, suggesting an endogenous yeast factor(s) can act to bind these DNA motifs in the Y1HGold background, independent of PnPf2. The dual-tandem copies of M1 and M2 were not auto-activated, but no increased activation of the AUR1-C reporter was observed in the presence of PnPf2. (PDF) [file ppat.1012536.s006.pdf]

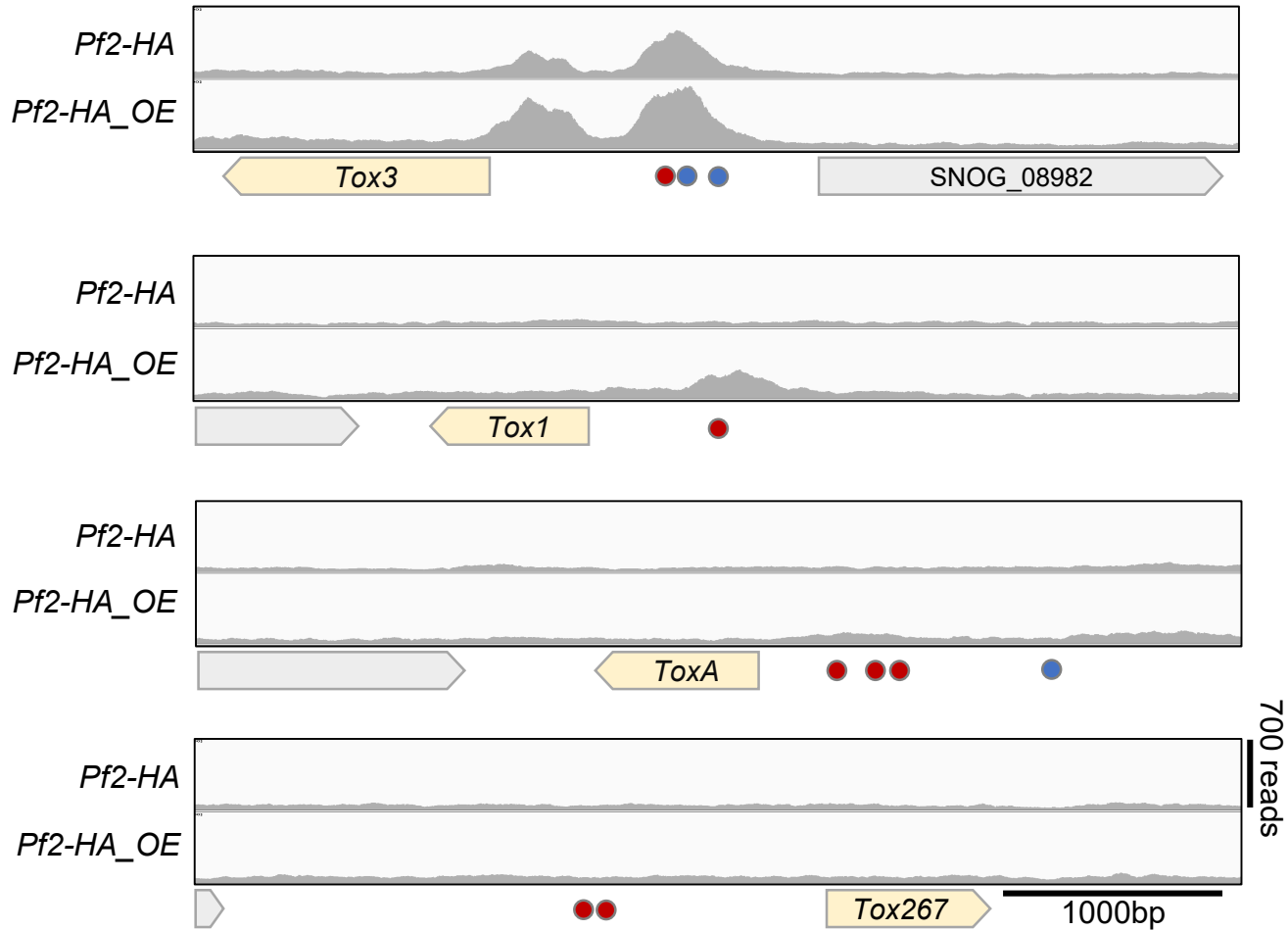

Supplement: S2 Fig — The Pf2-HA and Pf2-HA_OE ChIP-seq read peaks are presented at the Tox3, Tox1, ToxA and Tox267 promoters. Peak summits were evident in the Tox3 and Tox1 promoters. Red dots represent instances of the M1 motif (5’-RWMGGVCCGA-3’) and blue dots M2 (5’-CGGCSBYWYBKCGGC-3’). (PDF) [file ppat.1012536.s007.pdf]

**A**

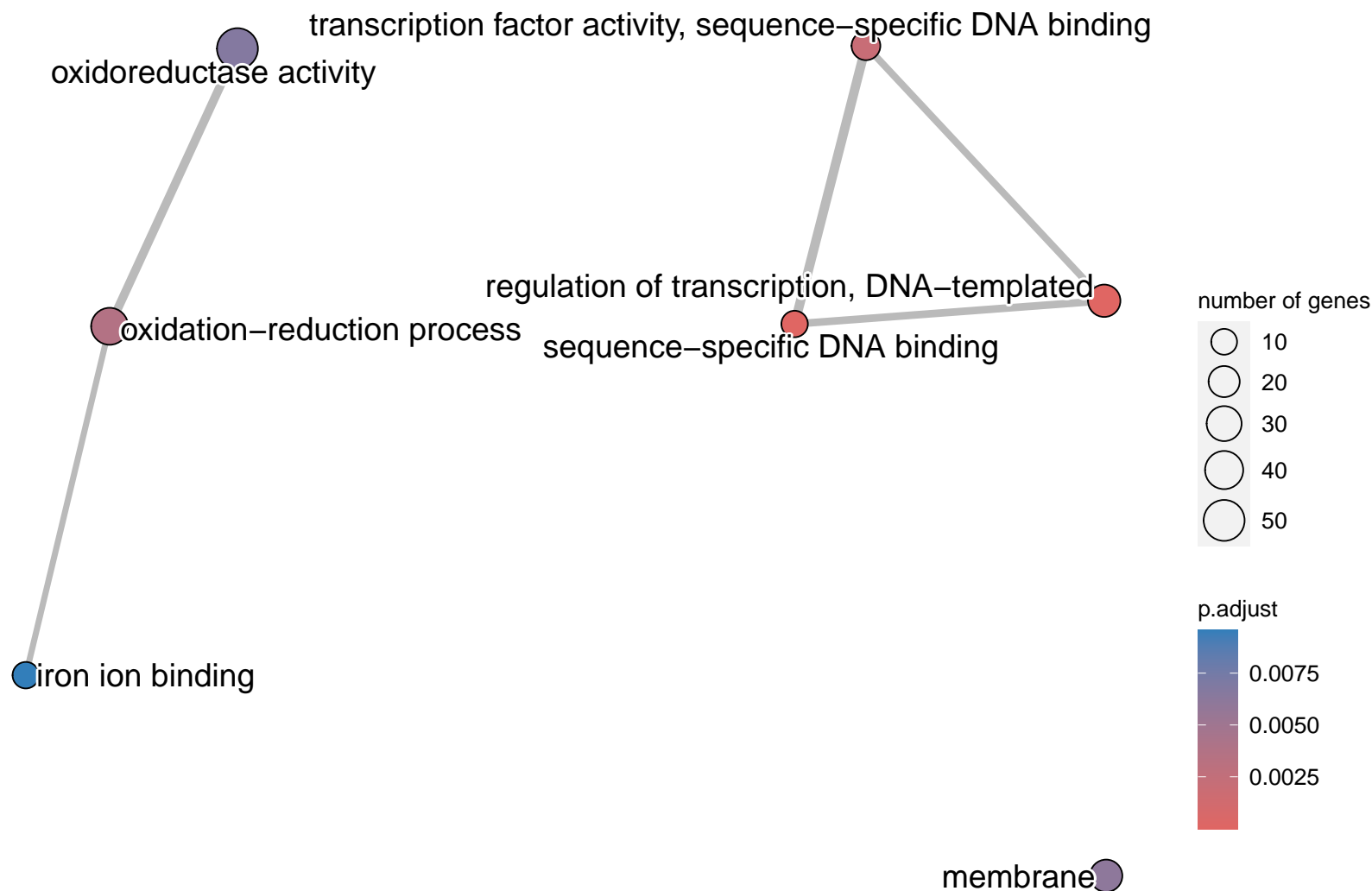

**B**

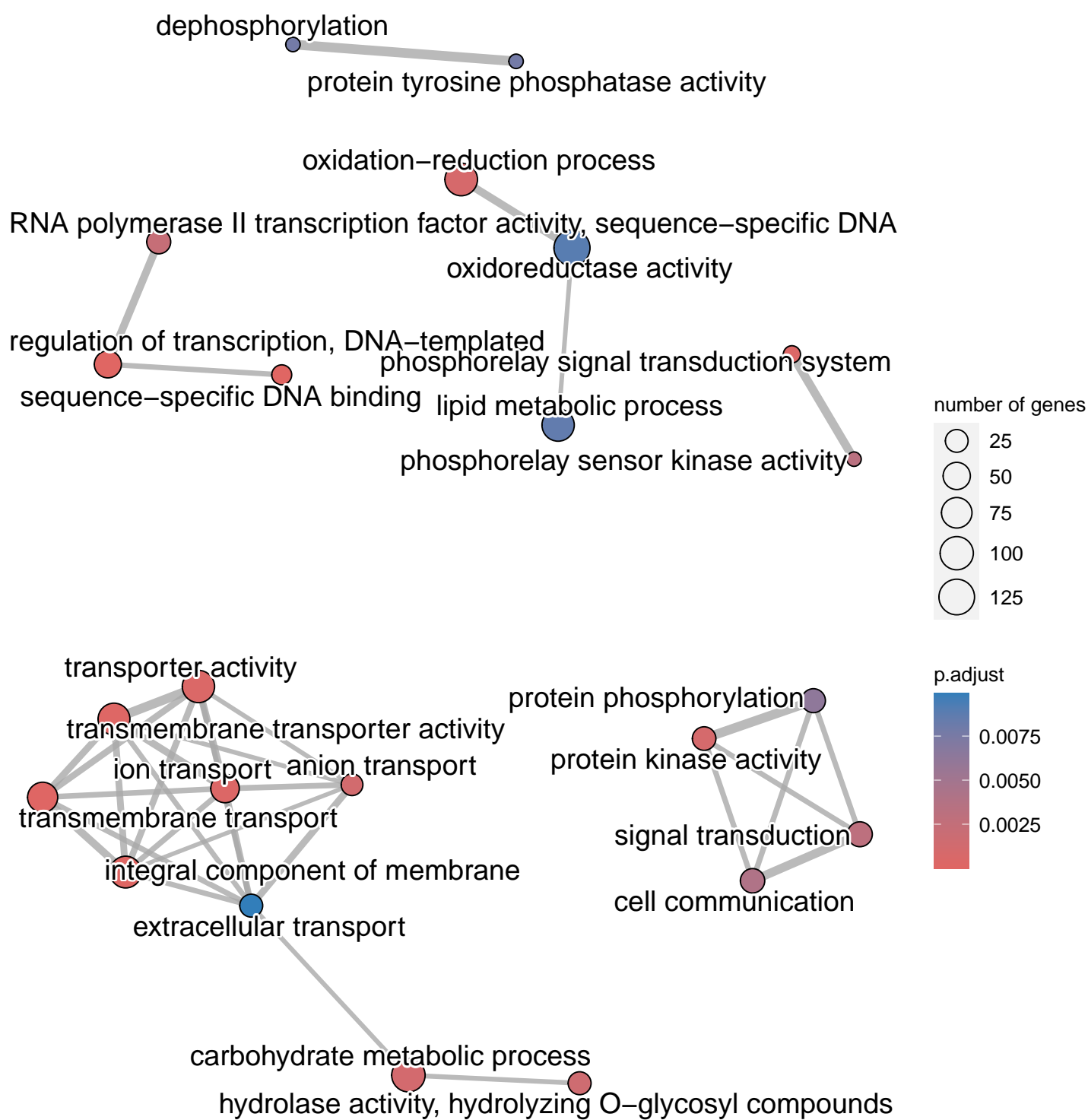

Supplement: S3 Fig — Original GO-enrichment analysis presented in Fig 3 including both A) the high-confidence PnPf2-targeted genes (total = 412) and B) all candidate PnPf2 targets (total = 1253). (PDF) [file ppat.1012536.s008.pdf]

# Hierarchical cluster analysis

## *Parastagonospora nodorum* gene expression

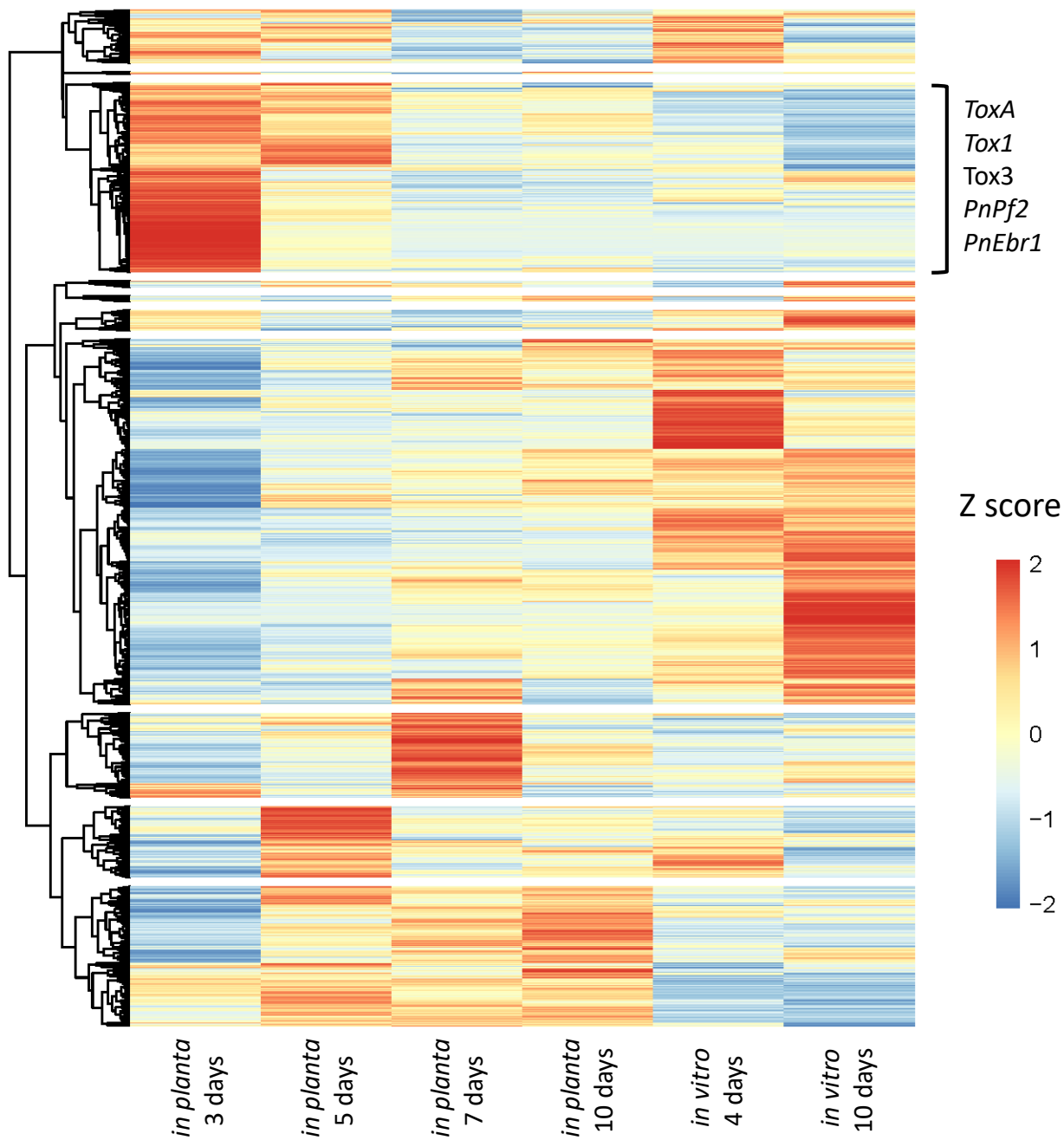

Supplement: S4 Fig — Clustering was based on microarray gene-expression data during infection (in planta) or axenic (in vitro) growth obtained from a previous study [77]. Genes were divided into the 10 most distant clusters to identify genes co-expressed with PnPf2, ToxA, Tox1 and Tox3, which included the Zn2Cys6 transcription factor PnEbr1 (SNOG_03037) therefore investigated in this study. (PDF) [file ppat.1012536.s009.pdf]
